# Exogenous abscisic acid treatment regulates protein secretion in sorghum cell suspension cultures

**DOI:** 10.1080/15592324.2023.2291618

**Published:** 2023-12-15

**Authors:** Dakalo Muthego, Sellwane J. Moloi, Adrian P. Brown, Tatenda Goche, Stephen Chivasa, Rudo Ngara

**Affiliations:** a Department of Plant Sciences, University of the Free State, Phuthaditjhaba, South Africa; b Department of Biosciences, Durham University, Durham, UK; c Department of Crop Science, Bindura University of Science Education, Bindura, Zimbabwe

**Keywords:** Sorghum, cell suspension cultures, ABA, extracellular matrix, protein secretion, secretome, total soluble protein, iTRAQ, drought stress, gene expression

## Abstract

Drought stress adversely affects plant growth, often leading to total crop failure. Upon sensing soil water deficits, plants switch on biosynthesis of abscisic acid (ABA), a stress hormone for drought adaptation. Here, we used exogenous ABA application to dark-grown sorghum cell suspension cultures as an experimental system to understand how a drought-tolerant crop responds to ABA. We evaluated intracellular and secreted proteins using isobaric tags for relative and absolute quantification. While the abundance of only ~ 7% (46 proteins) intracellular proteins changed in response to ABA, ~32% (82 proteins) of secreted proteins identified in this study were ABA responsive. This shows that the extracellular matrix is disproportionately targeted and suggests it plays a vital role in sorghum adaptation to drought. Extracellular proteins responsive to ABA were predominantly defense/detoxification and cell wall-modifying enzymes. We confirmed that sorghum plants exposed to drought stress activate genes encoding the same proteins identified in the *in vitro* cell culture system with ABA. Our results suggest that ABA activates defense and cell wall remodeling systems during stress response. This could underpin the success of sorghum adaptation to drought stress.

## Introduction

The phytohormone abscisic acid (ABA) regulates various physiological processes during plant growth and development, and in response to biotic and abiotic stresses.^1–6^ Although primarily regarded as a plant hormone, Olds *et al*.^5^ reviewed other roles of ABA in bacteria, protozoa, fungi, marine sponges, insects, and animals. As a plant stress hormone, ABA biosynthesis and accumulation are triggered by environmental stresses such as drought, salinity, and cold, which expose plants to osmotic stress.^1,7–9^ It then functions as a root-to-shoot long-distance signal coordinating plant responses to soil drying, such as stomatal closure for reducing transpiration, and restructuring plant growth.^7–10^ Upon relief from stress, ABA is either catabolized via hydroxylation by cytochrome P450 enzymes with ABA-8’-hydroxylase activity or conjugated to glucose by ABA glucosyltransferases to form the physiologically inactive ABA-glucosyl ester.^4,11^ Consequently, the biologically active ABA content reverts to pre-stress levels for restoration of normal metabolism.^4,9^

ABA binding to its receptor complex Pyrabactin Resistance 1/PYR1-Like/Regulatory Component of ABA Receptor (PYR/PRL-1/RCAR) abolishes the inhibitory effects of type 2 protein phosphatases (PP2C) on kinases.^3,12^ This triggers autophosphorylation of sucrose non-fermenting 1 (SnF1)-related protein kinases (SnRKs), which phosphorylate downstream target transcription factors to activate stress gene expression.^3,12,13^ The *cis*-acting element ABA-responsive element (ABRE) and the basic-domain leucine zipper (bZIP)-type ABRE-binding protein/ABRE-binding factors (AREB/ABFs) transcription factors act as positive regulators of the ABA signaling.^13–15^ ABA-independent drought stress-adaptive responses are also activated via interaction of the dehydration-responsive element (DRE)/C-repeat (CRT) *cis*-acting element with DRE-binding (DREB) transcription factors.^13,14^ Elaborate crosstalk between ABA-dependent and ABA-independent pathways^13,16^ leads to robust adaptive responses to maintain cellular homeostasis and survive stress.

Dehydration-induced gene expression leads to a change in the proteome and metabolome, with notable enrichment of molecules with protective functions against the primary and secondary effects of drought stress.^13^ For example, enzymatic and non-enzymatic antioxidants, osmoprotectants, late embryogenesis abundant proteins, and molecular chaperones accumulate as an effective countermeasure to osmotic stress.^13,14,16^ A key question emerging from this research is why most plant species remain sensitive to drought stress when they can activate these molecular responses. Much of this research has been conducted using drought-sensitive Arabidopsis.^17^ Analysis of drought-tolerant species, such as sorghum,^18,19^ could lead to new insights into the molecular signals that underpin field success against drought stress.

A variety of “omics” studies of diverse plant species have investigated expression changes of ABA-regulated transcripts,^20–25^ total soluble proteins (TSP),^24,26–31^ phosphoproteins^32–36^ and metabolites^27,31^ following exogenous ABA treatment with or without abiotic stress factors such as osmotic stress and high salinity. Phosphoproteomic analyses have also confirmed the central role of kinase activities in regulating ABA-responses. For example, an increase in phosphorylation states of members of the SnRK2s, particularly SnRK2.2, SnRK2.3 and SnRK2.6, several transcription factor families, including bZIP and multiple downstream proteins of various functions have been reported following exogenous ABA treatment.^32–36^ Likewise, the observed increased^36^ and decreased^33^ phosphorylation states of various aquaporins in response to ABA treatment could have implications in water transport across membranes under various environmental stresses. Undoubtedly, these “omics” studies continue to advance our understanding of ABA-regulated molecular responses.

However, limited proteomics studies have investigated the effects of exogenous ABA treatment on protein secretion,^37–39^ yet extracellular matrix (ECM) proteins play essential roles during plant development and stress response.^40–43^ Nevertheless, several plant proteomic studies have investigated secretome responses to osmotic, heat and high salinity stresses in diverse plant species,^42,44,45^ and the critical roles of these ECM proteins in stress response are unquestionable. Many of these studies have also utilized plant cell suspension cultures due to the inherent advantages of such model systems in secreted protein analyses.^42,44^ Therefore, this study investigated the differential expression profiles of total soluble and secreted proteins in sorghum cell suspension cultures following ABA treatment.

## Materials and Methods

### ABA treatments of sorghum cell suspension cultures

White sorghum (*Sorghum bicolor*) cell suspension cultures^46^ were used in this study. The cell suspension cultures were subcultured and maintained as described previously.^46,47^ All treatments were carried out on 8-day-old cell cultures, which corresponded to the mid-log phase.^47^ On day 8 post subculture, four biological replicate flasks were split into two 30 mL subcultures each for the control and ABA treatment to account for technical variation between samples. For ABA treatment, the cell suspension cultures were treated with a final concentration of 100 μM ABA using a filter-sterilized 0.1 M ABA (Catalog No. A1049, Sigma Aldrich, Saint Louis, USA) stock solution prepared in 70% methanol. The control cells were spiked with an equivalent volume of filter-sterilized 70% methanol. Cells of both treatment groups were incubated for 72 h during which cell viability^48,49^ and fresh/dry weight growth measurements were taken. The 0-h time point denotes the time immediately after treatment was imposed. For both cell viability and growth measurements, four biological replicate cell cultures were used for each treatment.

### Total soluble and secreted protein extraction

Four biological replicate control and ABA-treated cell cultures were harvested at 72 h post treatment for total soluble and secreted protein extraction as previously described.^50^ Briefly, cell suspension cultures were filtered through four layers of Miracloth to separate the cells from the medium. The cells were subsequently washed with sterile distilled water and stored at −80°C, while the medium was centrifuged at 2 500 × *g* for 10 minutes to collect the cell-free supernatant. Total soluble protein (TSP) was extracted from homogenized cells, while secreted proteins were extracted from the cell-free culture medium by acetone precipitation and centrifugation. The resultant protein pellets were solubilized in appropriate volumes of extraction buffer (7 M urea, 2 M thiourea, 4% (w/v) 3-(3-cholamidopropyl)dimethylammonio)-1-propanesulfonate) with vigorous vortexing overnight.^50^ The extracted TSP and secreted proteins were quantified and prepared for isobaric tags for relative and absolute quantitation (iTRAQ) and tandem mass spectrometry analysis. All protocols for iTRAQ labeling and liquid chromatography-tandem mass spectrometry (LC-MS/MS) are as described in previous publications from our groups.^48,50–52^

### iTRAQ labelling, LC-MS/MS, protein identification, and quantification

Aliquots of 12.5 μg protein from each sample were processed for labeling using an iTRAQ Reagent-Multiplex Buffer Kit (AB Sciex, Redwood City, CA, USA) following the manufacturer’s instructions. The protein samples were then digested with trypsin overnight^51^ and peptides subsequently labeled using an 8-plex iTRAQ reagent kit (AB Sciex) according to the manufacturer’s instructions. The 4-replicate control samples of TSP and secreted protein fractions were separately labeled with iTRAQ tags 113, 114, 115 and 116, while ABA-treated samples were labeled with tags 117, 118, 119 and 121. This gave rise to two separate iTRAQ experiments each with the control and ABA-treated samples of each proteome. After labeling, all TSP samples were pooled (across the 8 tags) as was also done for the secreted samples. The pooled iTRAQ-labeled samples were subsequently cleaned-up using hydrophobic interaction chromatography (HILIC) solid phase extraction (SPE) cartridges (PolyLC Inc., Columbia, MD, USA) as described previously.^52^ Then LC-MS/MS and mass spectrometric analyses were conducted on peptides originating from 5 μg of sample following detailed protocols described in Goche *et al*.^52^ LC-MS/MS was conducted using a Triple TOF 6600 mass spectrometer (AB Sciex) linked to an Eksigent 425 LC system via a Sciex Nanospray III source (AB Sciex). The acquisition of mass spectrometer data was done using the AB Sciex Analyst TF 1.7.1 instrument control and data processing software.

Protein identification and relative quantification were conducted using the detailed protocol described in Goche *et al*.^52^ with minor modifications. The raw.wiff data files were processed against the UniProt protein sequences of *Sorghum bicolor* only (downloaded in May 2018) using the AB Sciex ProteinPilot 5.01 version 4895 software with the Paragon Algorithm 5.0.1.0.4874. The raw protein identification data were exported from ProteinPilot to Microsoft® Excel version 16.16.27 for manual data-handling and filtering. All duplicate proteins and those identified based on a single peptide were manually removed from the dataset, giving rise to 707 and 257 positively identified proteins in the TSP and secreted protein fractions, respectively. For each iTRAQ experiment, the relative quantification of the ABA-responsive proteins was generated as a ratio of each protein relative to the 113-tagged control sample. Then an average ratio of each protein was computed across all four biological replicate samples. Thereafter, a Student’s *t*-test at *p* ≤.05 was used to calculate the statistical significance in the ratios.

### Drought treatments and analysis of gene expression

Drought-tolerant SA1441 and drought-susceptible ICSB338 sorghum lines were used in drought stress experiments as previously described.^52^ Briefly, seeds were germinated and transplanted into F2 + sand mixture (ICL Ltd., Ipswich, UK) in 216 cm^3^ pots incubated in a 16 h-photoperiod at 25–30°C. Plants were allowed to grow with adequate watering to the developmental stage with three fully expanded leaves and an emerging fourth leaf. Watering was stopped and root tissues harvested 12 days later for RNA extraction. Spectrum Plant Total RNA Kit (Merck Life Science, Dorset, UK) was used for total RNA extraction following the manufacturer’s instruction and cDNA synthesis used the GoScript Reverse Transcriptase System (Promega, Southampton, UK) according to a protocol provided by the manufacturer. In this experiment, three biological replicate RNA extracts were prepared per sorghum line and treatment for use in cDNA synthesis.^52^

Quantitative reverse transcription – polymerase chain reaction analysis was conducted as described by Goche *et al*.^52^ All reactions were carried out for three biological replicates, each with three technical replicates on a Corbett Rotor-Gene 6000 (Qiagen, Cambridge, UK) following the thermal cycling conditions described by Goche *et al*.^52^ The REST2009 software version 2.0.13 (Qiagen) was used for data analysis, while the Student’s *t*-test at *p* ≤.05 was used to compare the gene expression fold-change. The following genes and primer sequences were used: *PR4 (Pathogenesis-related protein-4*, SORBI_3005G169300) 5’-GCTACCAGATGGGTCACCTC-3’and 5’-TGATACGCTCCTCATGTCGC-3’; *PRX3* (*Peroxidase-3*, SORBI_3002G391300) 5’-AAGGCCATGGTGAAGATGGG-3’ and 5’-GGCAGTTGGTCCTGATCTCC-3’; *PRX9* (*Peroxidase-9*, SORBI_3003G152100) 5’-GCGCGTGTGCATGAGTATTG-3’ and 5’-GTGCGCAACAAACAACAAGC-3’; *GH19–1* (*Glycoside hydrolase family 19–1*, SORBI_3006G132300) 5’-TGTTGCCTCGAAACAGTGTG-3’and 5’-AGCTGCAACCGTAAACTTTG-3’; *GH19–2* (*Glycoside hydrolase family 19–2*, SORBI_3006G132400) 5’-CAGTCCTTGGATGGCTCGTC-3’ and 5’-GGTGCAACAAACAGGCTCAG-3’, *GH9* (*Glycoside hydrolase family 9*, SORBI_3003G015700) 5’-AGCAGCTATAGTGTGTTGCTTG-3’ and 5’-GCTAAATGAACAAGACGGTCCAG-3’; *NLTP* (*Plant non-specific lipid-transfer protein/Par allergen*, SORBI_3008G030900) 5’- CTCCGCACCCTCAGCAG-3’and 5’-CGTTCTTGAGGCAGTTGCAG-3’; *LRRP* (*Leucine-rich repeat-containing N-terminal plant-type domain-containing protein*, SORBI_3005G126200) 5’-CGGTTCCATCGGAAGTCCTC-3’ and 5’-CATGCAGTCTTCAGCGCATC-3’; *GELP* (*GDSL lipase/esterase-like plant*, SORBI_3010G044500) 5’-GATTGCAACCAGCTTAGCCC-3’ and 5’-GCAAAGACCAAAGAGGGTCC-3’. Two gene were used as constitutive reference controls:^52^ *eIF4A* (Sb04g003390) 5’-GATGAGATGCTCTCCCGTGG-3’and 5’-TGATCTCTAGGGCCTCTGGG-3’; uncharacterized protein (Sb03g038910) 5’-TCCTGAAGCATCTTTCCCTCC-3’ and 5’-ACAGCCTGATTAGTTGGGGG-3’. All gene specific primers were designed using the National Centre for Biotechnology (NCBI) Primer-BLAST software.

### Bioinformatics analysis

Gene ontology analysis and protein family names were retrieved from the UniProt^53^ and Interpro^54^ databases, respectively. The signal peptide predictions were conducted on the SignalP 6.0 server.^55^

## Results and discussion

### Plant cell suspension cultures are a useful resource for studying ABA-response

ABA is a multifunctional phytohormone,^1–4^ and its role in plant stress response has been extensively studied and reviewed.^3,14,16,56,57^ Numerous proteomics studies have implicated ABA-responsive total soluble proteins^24,26–29,31^ and phosphoproteins^32,33,35,36^ in signaling, regulatory and protective functions during stress adaptation.^14,16^ To our knowledge, however, similar investigations on the effects of exogenous ABA on secretory proteins are minimal,^37,39^ yet the secretome is essential during plant cell growth, development, and stress response.^40–43^

The secretome consists of proteins that are secreted into the ECM of plant cells.^42^ Some reviews^42,44^ have summarized secretome studies of various plant species in response to biotic and abiotic stresses using whole plants or cell suspension cultures. In the current study, we used sorghum cell suspension cultures in line with other secretome studies that we have conducted.^48,51^ The utility of cell suspension cultures in ABA responses of total soluble proteins^24,27^ and the secretome^37^ has also been tested and validated in Arabidopsis (*Arabidopsis thaliana*),^24^ rice (*Oryza sativa*)^27^ and wheat (*Triticum aestivum*).^37^ Therefore, our white sorghum cell suspension cultures provided an experimental resource for comparing the impact of exogenous ABA on expression profiles of intracellular versus extracellular proteins.

Analysis of cellular metabolic activity (Figure S1) and fresh and dry weight (Figure S2) of sorghum cell cultures revealed no adverse effects of ABA treatment over the 72-h of exposure. The increase in cell weight from 0–72 h (Figure S2) reflected cell culture growth. Thus, the level of ABA applied, and timing of cell harvest did not compromise either cell vitality or growth, thus enabling the identification of intracellular and extracellular proteins recruited in ABA-dependent responses.

### iTRAQ reveals the selective nature of protein secretion in ABA-treated sorghum cell suspension cultures

The TSP fraction was extracted from the cells, while the secreted proteins were extracted from the growth medium prior to digestion with trypsin, iTRAQ labeling and LC-MS/MS analysis. A total of 707 TSP and 257 secreted proteins were positively identified based on at least two matching peptides, and the peptide information is listed in Tables S1 and S2. The observed differences in the proportions of positively identified proteins under untreated conditions possibly reflect differences in protein diversity and function of the two cellular compartments, and the specialized and selective nature of protein secretion. Although all proteins are synthesized intracellularly,^58^ only a subset of these proteins is secreted and enriched into the ECM for specific functions.^40–44^

The iTRAQ data were subsequently analyzed using a Student’s *t*-test at a 5% significance level to identify the ABA-responsive proteins. In total, 46 (~7%) TSP and 82 (~32%) secreted proteins were ABA-responsive (*p* ≤.05), as summarized in Table 1, while their iTRAQ quantitation data are given in Tables S3 and S4. A large proportion of the combined subset of ABA-responsive proteins amounting to 68%, were uncharacterized (Tables S5 and S6), as observed in other sorghum iTRAQ datasets^48,51,52^. This calls for increased experimental validation studies^59–61^ of sorghum genes and proteins for improved functional annotations.

**Table 1.** Summary list of sorghum protein counts obtained after iTRAQ and LC-MS/MS analysis.

Proteome	Positively identified proteins	ABA-responsive	Up-regulated	Down-regulated
TSP	707	46	30	16
Secreted	257	82	47	35

TSP – total soluble protein.

### The extent of ABA effects on metabolism covers nearly all intracellular and extracellular compartments

We then collected bioinformatics data on signal peptide predictions, Gene Ontology (GO) terms, and protein family names to assist in assigning putative cellular locations and biological functions to these proteins (Tables S5 and S6). However, due to the extensive list of ABA-responsive proteins obtained in this study using a Student’s *t*-test at *p* ≤.05 (Tables S5 and S6), we have shortened this list for illustrative purposes in Tables 2 and 3 by only showing ABA-responsive proteins based on a more stringent probability value (*p* ≤.01). Nevertheless, all results presentations and discussions in this study are based on the entire ABA-responsive protein selection at *p* ≤.05 (Tables S5 and S6).

**Table 2.** List of ABA-responsive total soluble proteins of white sorghum cell suspension cultures at 1% significance level.

N^a^	Accession^b^	Protein name	Ratio^c^	SD^d^	p-value^e^	MW (kDa)^f^	SP^g^	Cellular component^h^	Biological process^i^	Molecular function^j^	Protein family^k^
**Metabolism**											
81	A0A194YT53	Uncharacterized protein OS=*Sorghum bicolor* GN=SORBI_3004G345800	1.41	0.15	2.70E–03	48.17	-	Peroxisome	Fatty acid beta-oxidation	Acyltransferase activity	Thiolase
340	C5XI18	S-adenosylmethionine synthase OS=*Sorghum bicolor* GN=SORBI_3003G140000	−1.37	0.05	1.07E–03	43.26	-	Cytoplasm	S-adenosylmethionine biosynthetic process	Methionine adenosylmethionine activity	S-adenosylmethionine synthetase
456	C5Z513	Uncharacterized protein OS=*Sorghum bicolor* GN=SORBI_3010G171800	−1.31	0.09	6.98E–03	63.13	+	Extracellular region	None	L-ascorbate oxidase activity	L-ascorbate oxidase, plants
596	C5XRZ8	Uncharacterized protein OS=*Sorghum bicolor* GN=SORBI_3004G296800	1.28	0.13	8.89E–03	25.76	-	None	Nitrogen compound metabolic process	Hydro-lyase activity	Aconitase A/isopropylmalate dehydratase small subunit swivel domain-containing
**Defence/Detoxification**											
132	C5WWQ2	Uncharacterized protein OS=*Sorghum bicolor* GN=SORBI_3001G342600	−1.58	0.07	9.51E–03	64.78	-	None	Cellular oxidant detoxification	Thioredoxin-disulfide reductase activity	Thioredoxin domain-containing protein
**Signal transduction**											
47	C5WMM0	Uncharacterized protein OS=*Sorghum bicolor* GN=SORBI_3001G400900	1.47	0.23	8.53E–03	17.06	-	Nucleus	Abscisic acid-activated signaling pathway	Abscisic acid binding	Bet v I type allergen
**Proteolysis**											
561	C5Z3R9	Proteasome subunit beta type OS=*Sorghum bicolor* GN=SORBI_3010G029400	1.30	0.04	6.25E–04	26.28	-	Proteasome complex	Proteolysis involved in protein catabolic process	Threonine-type endopeptidase activity	Peptidase T1A, Proteasome beta-subunit
**Cell wall modification**											
76	C5XKE9	Endoglucanase OS=*Sorghum bicolor* GN=SORBI_3003G015700	1.55	0.14	8.60E–04	69.78	+	Extracellular region	Carbohydrate metabolic process	Hydrolase activity, hydrolyzing O-glycosyl compounds	Glycoside hydrolase family 9
571	C5XVQ6	Glycosyltransferase OS=*Sorghum bicolor* GN=SORBI_3004G191000	1.33	0.12	4.93E–03	56.01	-	Cytoplasm	None	UDP-glycosyltransferase activity	UDP-glucuronosyl/UDP-glycosyltransferase
**Unclassified**											
117	C5XBP7	Uncharacterized protein OS=*Sorghum bicolor* GN=SORBI_3002G343600	2.10	0.14	5.53E–05	35.66	-	None	Specification of floral organ number	Enzyme inhibitor activity	Leucine-rich repeat-containing N-terminal plant-type domain-containing protein

^a^Protein number (N) assigned in ProteinPilot.

^b^Protein accession numbers obtained from the UniProt database searches against sequences of *Sorghum bicolor* only.

^c^Ratio represents the average fold-change (*n* = 4) in response to ABA relative to the control. A positive value indicates up-regulation, while a negative value indicates down-regulation.

^d^Standard deviation of the fold-changes (*n* = 4).

^e^Probability value obtained from a Student’s *t*-test comparing the fold changes between the ABA treatment and the control (*n* = 4).

^f^Theoretical molecular weight (MW) of each protein as predicted by the Expasy Compute pI/Mw tool on the UniProt database (https://uniprot.org).

^g^Signal peptide (SP) prediction results for each protein as determined by the SignalP 6.0 server (https://services.healthtech.dtu.dk/services/SignalP-6.0/). + indicated ^p^resence of a signal peptide, while – indicates absence of a signal peptide.

^h–j^Gene Ontology terms for each protein as collated from the UniProt database.

^k^Family name as predicted using the InterPro (http://www.ebi.ac.uk/interpro/). In cases where protein families are not predicted, functional domains are listed instead.

**Table 3.** List of ABA-responsive secreted proteins of white sorghum cell suspension cultures at 1% significance level.

N^a^	Accession^b^	Protein name	Ratio^c^	SD^d^	p-value^e^	MW (kDa)^f^	SP^g^	Cellular component^h^	Biological process^i^	Molecular function^j^	Protein family^k^
**Metabolism**											
191	C5Z4E5	Uncharacterized protein OS=*Sorghum bicolor* GN=SORBI_3010G044900	−1.68	0.09	6.64E–04	39.46	+	None	None	Hydrolase activity. acting on ester bonds	GDSL lipase/esterase-like. plant
208	A0A194YIA9	Uncharacterized protein OS=*Sorghum bicolor* GN=SORBI_3010G044500	2.92	0.44	2.22E–04	38.36	+	None	None	Hydrolase activity. acting on ester bonds	GDSL lipase/esterase-like. plant
**Defence/Detoxification**											
5	C5X5K6	Peroxidase OS=*Sorghum bicolor* GN=SORBI_3002G416700	−1.38	0.03	3.53E–03	32.41	+	Extracellular region	Response to oxidative stress	Peroxidase activity	Plant Peroxidase
6	C5WYQ4	Peroxidase OS=*Sorghum bicolor* GN=SORBI_3001G360400	−1.65	0.09	3.82E–04	34.75	+	Extracellular region	Response to oxidative stress	Peroxidase activity	Plant Peroxidase
26	C5XCE2	Uncharacterized protein OS=*Sorghum bicolor* GN=SORBI_3002G351400	1.63	0.27	6.06E–03	24.57	+	None	Defense response	None	Thaumatin family
29	C5XN52	Uncharacterized protein OS=*Sorghum bicolor* GN=SORBI_3003G331700	2.21	0.22	4.79E–05	23.88	+	None	Defense response	None	Thaumatin family
54	C5YM54	Uncharacterized protein OS=*Sorghum bicolor* GN=SORBI_3007G151300	−1.93	0.02	7.23E–03	22.73	+	Apoplast	None	Metal ion binding	Germin
142	C5YC92	Uncharacterized protein OS=*Sorghum bicolor* GN=SORBI_3006G018100	1.43	0.08	5.84E–05	24.97	+	Apoplast	None	Metal ion binding	Germin
161	C5YQ75	Peroxidase OS=*Sorghum bicolor* GN=SORBI_3008G010500	−1.85	0.11	2.75E–04	34.73	+	Extracellular region	Response to oxidative stress	Peroxidase activity	Plant Peroxidase
180	C5Y5D5	Uncharacterized protein OS=*Sorghum bicolor* GN=SORBI_3005G169300	2.68	0.58	1.40E–03	16.02	+	None	Defense response	RNA nuclease activity	Pathogenesis-related protein-4
183	A0A1B6QJR7	Peroxidase OS=*Sorghum bicolor* GN=SORBI_3001G189000	1.80	0.35	3.98E–03	43.29	-	Extracellular region	Response to oxidative stress	Peroxidase activity	Plant peroxidase
265	C5XIY0	Peroxidase OS=*Sorghum bicolor* GN=SORBI_3003G152000	−1.49	0.05	7.51E–03	37.65	+	Extracellular region	Response to oxidative stress	Peroxidase activity	Plant peroxidase
**Proteolysis**											
49	C5Y675	Uncharacterized protein OS=*Sorghum bicolor* GN=SORBI_3005G064200	−1.30	0.09	8.37E–03	44.46	+	None	Proteolysis	Aspartic-type endopeptidase activity	Aspartic peptidase A1 family
82	C5WVG9	Cysteine proteinase inhibitor OS=*Sorghum bicolor* GN=SORBI_3001G324800	−1.58	0.09	3.44E–03	14.38	+	None	Negative regulation of peptidase activity	Cysteine-type endopeptidase inhibitor activity	Cystatin
117	A0A1B6Q6M7	Cysteine proteinase inhibitor OS=*Sorghum bicolor* GN=SORBI_3003G327700	1.91	0.26	7.40E–04	14.89	+	None	Negative regulation of peptidase activity	Cysteine-type endopeptidase inhibitor activity	Cystatin
132	A0A1B6P5R2	Uncharacterized protein OS=*Sorghum bicolor* GN=SORBI_3009G009600	2.02	0.34	2.32E–03	16.56	-	None	Negative regulation of peptidase activity	Serine-type endopeptidase inhibitor activity	Proteinase inhibitor I13. potato inhibitor I
226	C5YNA1	Uncharacterized protein OS=*Sorghum bicolor* GN=SORBI_3007G172100	1.47	0.10	1.33E–03	40.26	+	Extracellular space	Proteolysis	Cysteine-type endopeptidase activity	Peptidase C1A
235	C5X0Y6	Uncharacterized protein OS=*Sorghum bicolor* GN=SORBI_3001G529700	1.70	0.14	3.77E–04	79.49	+	None	Proteolysis	Serine-type endopeptidase activity	Subtilisin-like protease
**Cell wall modification**											
3	C5XYP5	Uncharacterized protein OS=*Sorghum bicolor* GN=SORBI_3004G233700	−1.28	0.03	6.49E–04	84.26	+	None	Carbohydrate metabolic process	Hydrolase activity. hydrolyzing O-glycosyl compounds	Beta-D-xylosidase
13	C5Z8N0	Uncharacterized protein OS=*Sorghum bicolor* GN=SORBI_3010G118900	−1.56	0.06	2.95E–03	47.82	+	Plasma membrane	None	None	Fasciclin-like arabinogalactan protein
16	C5XKE9	Endoglucanase OS=*Sorghum bicolor* GN=SORBI_3003G015700	3.30	0.88	2.22E–03	69.78	+	Extracellular region	Carbohydrate metabolic process	Hydrolase activity. hydrolyzing O-glycosyl compounds	Glycoside hydrolase family 9
19	C5YBE9	Uncharacterized protein OS=*Sorghum bicolor* GN=SORBI_3006G132400	3.93	0.31	1.73E–06	28.56	+	None	Carbohydrate metabolic process	Chitinase activity	Glycoside hydrolase. family 19
22	C5Z8T4	Xyloglucan endotransglucosylase/hydrolase OS=*Sorghum bicolor* GN=SORBI_3010G246600	−1.47	0.06	6.01E–05	31.49	+	Extracellular region	Carbohydrate metabolic process	Hydrolase activity. hydrolyzing O-glycosyl compounds	Xyloglucan endotransglucosylase/hydrolase
25	C5XB38	Uncharacterized protein OS=*Sorghum bicolor* GN=SORBI_3002G055600	2.05	0.19	8.37E–05	33.65	+	Extracellular region	Carbohydrate metabolic process	Chitinase activity	GH18 domain-containing protein
50	C5X022	Uncharacterized protein OS=*Sorghum bicolor* GN=SORBI_3001G525000	−1.58	0.01	3.90E–04	49.22	+	None	Carbohydrate metabolic process	Hydrolase activity. hydrolyzing O-glycosyl compounds	Glycoside hydrolase. family 28
51	C5YBE8	Uncharacterized protein OS=*Sorghum bicolor* GN=SORBI_3006G132300	4.81	0.89	1.62E–04	28.28	+	None	Carbohydrate metabolic process	Chitinase activity	Glycoside hydrolase. family 19
64	A0A1B6Q838	Uncharacterized protein OS=*Sorghum bicolor* GN=SORBI_3003G422200	1.84	0.23	1.68E–03	34.95	+	None	Carbohydrate metabolic process	Hydrolase activity. hydrolyzing O-glycosyl compounds	Glycoside hydrolase family 17
74	C5Y5V0	Uncharacterized protein OS=*Sorghum bicolor* GN=SORBI_3005G177600	1.40	0.07	8.58E–03	32.94	+	Extracellular region	Carbohydrate metabolic process	Chitinase activity	GH18 domain-containing protein
90	C5WSE5	Uncharacterized protein OS=*Sorghum bicolor* GN=SORBI_3001G300400	1.66	0.27	4.82E–03	31.85	+	Extracellular region	Cell wall organization	None	Expansin
171	C5Y5U9	Uncharacterized protein OS=*Sorghum bicolor* GN=SORBI_3005G177500	1.24	0.07	6.06E–03	33.36	+	Extracellular region	Carbohydrate metabolic process	Chitinase activity	GH18 domain-containing protein
188	C5XHS1	Uncharacterized protein OS=*Sorghum bicolor* GN=SORBI_3003G422000	2.58	0.43	5.39E–03	35.69	+	None	Carbohydrate metabolic process	Hydrolase activity. hydrolyzing O-glycosyl compounds	Glycoside hydrolase family 17. plant
225	C5XIT5	Pectinesterase OS=*Sorghum bicolor* GN=SORBI_3003G148300	−1.53	0.06	5.68E–03	59.38	+	Extracellular region	Cell wall organization	Pectinesterase activity	Pectinesterase
**Cellular transport**											
105	C5YRL0	Non-specific lipid-transfer protein OS=*Sorghum bicolor* GN=SORBI_3008G030700	2.41	0.54	2.19E–03	12.05	+	None	Lipid transport	Lipid binding	Plant nonspecific lipid-transfer protein/Par allergen
**Unclassified**											
42	C5XBP7	Uncharacterized protein OS=*Sorghum bicolor* GN=SORBI_3002G343600	1.82	0.35	4.37E–03	35.66	+	None	Specification of floral organ number	Protein binding	Leucine-rich repeat-containing N-terminal plant-type domain-containing protein
81	C5XYB4	Uncharacterized protein OS=*Sorghum bicolor* GN=SORBI_3004G229300	2.36	0.28	3.93E–03	34.29	+	Extracellular space	None	None	Protein EXORDIUM-like
137	C5Z6Y0	Uncharacterized protein OS=*Sorghum bicolor* GN=SORBI_3010G088700	2.73	0.22	1.35E–05	34.48	+	Extracellular space	None	None	Protein EXORDIUM-like
173	C5YI64	Uncharacterized protein OS=*Sorghum bicolor* GN=SORBI_3007G198000	−1.56	0.14	7.04E–03	25.17	+	Membrane	None	None	DOMON domain-containing protein
281	C5Y2R8	Uncharacterized protein OS=*Sorghum bicolor* GN=SORBI_3005G126200	−3.34	0.10	9.82E–03	26.58	+	None	None	Protein binding	Leucine-rich repeat-containing N-terminal plant-type domain-containing protein

^a^Protein number (N) assigned in ProteinPilot.

^b^Protein accession numbers obtained from the UniProt database searches against sequences of *Sorghum bicolor* only.

^c^Ratio represents the average fold-change (*n* = 4) in response to ABA relative to the control. A positive value indicates up-regulation, while a negative value indicates down-regulation.

^d^Standard deviation of the fold-changes (*n* = 4).

^e^Probability value obtained from a Student’s *t*-test comparing the fold changes between the ABA treatment and the control (*n* = 4).

^f^Theoretical molecular weight (MW) of each protein as predicted by the Expasy Compute pI/Mw tool on the UniProt database (https://uniprot.org).

^g^Signal peptide (SP) prediction results for each protein as determined by the SignalP 6.0 server (https://services.healthtech.dtu.dk/services/SignalP-6.0/). + indicated presence of a signal peptide, while – indicates absence of a signal peptide.

^h^ ^– j^Gene Ontology terms for each protein as collated from the UniProt database.

^k^Family name as predicted using the InterPro (http://www.ebi.ac.uk/interpro/). In cases where protein families are not predicted, functional domains are listed instead.

**Figure 1.**
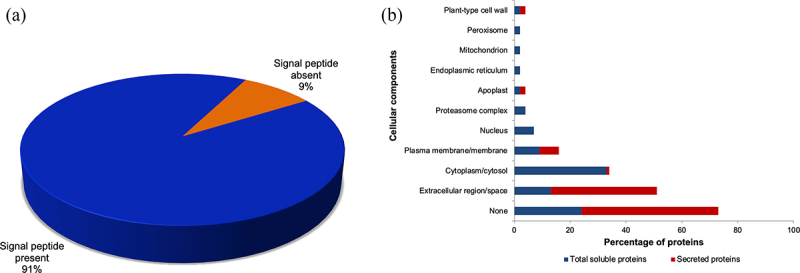
Predictions of signal peptides and cellular locations of ABA-responsive proteins of sorghum cell suspension cultures. (a) signal peptide predictions for ABA-responsive secreted proteins were done using the SignalP 6.0 server. (b) gene ontology terms for cellular components of ABA-responsive secreted and total soluble proteins were retrieved from the UniProt database.

Newly synthesized secretory proteins may be targeted for secretion via the conventional endoplasmic reticulum (ER)/Golgi-mediated pathway.^62,63^ Here, N-terminal signal peptides direct proteins to the ER and Golgi apparatus for posttranslational modifications before secretion via Golgi vesicles.^62,63^ However, some secreted proteins are leaderless, signal peptide-lacking proteins and may be trafficked into the ECM by other means that bypass the Golgi apparatus.^62,64–66^ Accordingly, signal peptide prediction results using the primary sequences of the ABA-responsive secretome (Table S6) on SignalP 6.0^55^ revealed that most of the ABA-responsive secreted proteins of sorghum had predictable signal peptides (91%), while the rest were leaderless (Figure 1a; Table S6). These results are comparable to a previous sorghum secretome, where 84% of the heat-responsive secreted proteins possessed signal peptides,^48^ but much higher than the 54% observed in an osmotic-stress study.^51^ Nonetheless, the results indicate that exogenous ABA triggers protein secretion in sorghum cell suspension cultures, as previously reported in wheat.^37^

Examples of N-terminal signal peptide-containing secreted proteins identified in this study include peroxidases, phytocyanins, GDSL lipase/esterases, thaumatins, germins, pathogenesis-related proteins, various proteases and their inhibitors, expansins, pectinesterases, lipid transfer proteins, leucine-rich repeat-containing proteins and several members of the glycoside hydrolase superfamily (Table S6). As reviewed by Alexandersson *et al*. ^44^ many of these proteins are members of secreted protein families under diverse environmental stresses in different plant species. Examples of leaderless ABA-responsive sorghum secreted proteins identified include members of the glycoside hydrolase family 31, UDP-glucuronosyl/UDP-glycosyltransferase, plant peroxidase, superoxide dismutase (SOD) and proteinase inhibitor II3, potato inhibitor I families (Table S6). The leaderless plant peroxidases with accessions A0A1W0W7I8 and A0A1B6QJR7 were associated with the plant cell wall and extracellular region, respectively (Table S6), thus pointing to a secretory location. On the other hand, extracellular plant SODs are known to lack signal peptides and have been reported in various plant secretome studies under pathogenic attack^64^ osmotic^51^ and heat^48^ stresses. These signal peptide-lacking proteins of sorghum now form part of the growing list of leaderless secretory proteins in plants^62,65^ that await cellular localization studies.

The signal peptide predictions of the ABA-responsive sorghum secretome (Figure 1a) were supported in part by the cellular component GO terms obtained (Figure 1b). For instance, the terms extracellular region and extracellular space were highly enriched in the secreted proteins versus the cytoplasm and cytosol locations in the ABA-responsive TSP (Figure 1b). In addition, the nucleus, proteasome complex, endoplasmic reticulum, mitochondrion, and peroxisome were exclusively identified in the ABA-responsive TSP fraction (Figure 1b). The diversity of subcellular localizations observed in the protein lists (Figure 1b; Tables S5 and S6) indicate that the extent of ABA effects on metabolism covers nearly all cell compartments. This confirms the role of ABA as a switch from normal growth to stress metabolism.

### Exogenous ABA application modulates specific stress-related processes in the ECM

**Figure 2.**
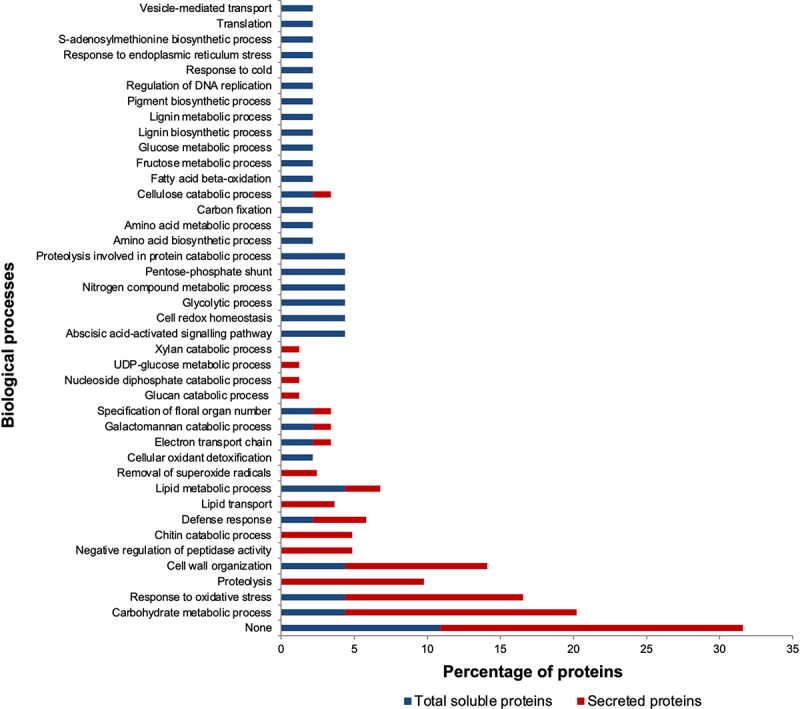
Biological processes of ABA-responsive secreted and total soluble proteins of sorghum cell suspension cultures. Gene Ontology terms for biological processes were retrieved from the UniProt database.

**Figure 3.**
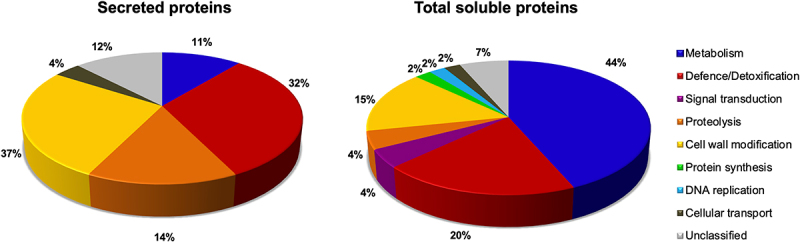
Putative functional groupings of the intracellular and extracellular ABA-responsive proteins of sorghum cell suspension cultures.

To understand the effects of ABA on ECM proteins, we used GO data of biological processes (Figure 2) and protein family names (Tables S6) to assign putative functions to the ABA-responsive secreted proteins (Figure 3). We then compared the functional groupings of the 46 ABA-responsive intracellular and 82 ECM proteins (Table 1) to assess whether ABA exerts a differential impact on ECM versus intracellular proteins (Figures 2 and 3; Tables S5 and S6). The results revealed that ABA modulates a variety of cellular processes in both proteomes (Figures 2 and 3). However, ABA effects on the secretome were primarily targeted toward defence/detoxification (32%), cell wall modification (27%), proteolysis (15%), metabolism (11%) and cellular transport (4%) (Figure 3). In contrast, the ABA-responsive TSP was mainly associated with metabolism (43%), defence/detoxification (20%), cell wall modification (15%), proteolysis (4%), and cellular transport (2%). In addition, signal transduction (4%), protein synthesis (4%) and DNA replication (2%) functional categories were unique to the TSP. Furthermore, while the intracellular fraction only had 46 ABA-responsive proteins compared to 82 ECM proteins (Table 1), the TSP represented greater diversity in protein families (Table S5), biological processes (Figure 2) and functional groupings (Figure 3). These results point to the diverse nature and function of total soluble proteins that are responsive to ABA, compared to a selective ABA-responsive secretome with specialized functions.

#### Plant defence and detoxification

Plant defense and reactive oxygen species (ROS) detoxification systems are vital for the survival of plants under biotic and abiotic stresses.^67–70^ In this study, the ABA-responsive secretome was dominated by defense/detoxification-related proteins (32%) (Figure 3), and most were up-regulated (Table S6). Examples include plant peroxidases, thaumatins, germins, SODs and pathogenesis-related (PR) proteins with RNA nuclease (e.g. PRP-4) and chitinase (e.g. glycoside hydrolase family 18 and 19) activities (Table S6). These proteins are known for their protective roles in plants against pathogen invasion and abiotic stress-induced oxidative damage.^67,69–72^ For instance, plant cells under pathogen attack generate an oxidative burst at the site of infection, which is mediated by germins, SODs and peroxidases.^70,73^ Apoplastic peroxidases also generate hydrogen peroxide, which functions as a ROS signal for stress-induced gene expression^72,74^ and in cell wall stiffening to prevent pathogen invasion.^70,73^ Other plant secretome studies have also identified various ROS antioxidant enzymes in response to heat,^48^ osmotic,^51^ dehydration,^75^ and salt^76^ stresses. About 20% of the identified ABA-responsive TSP were related to ROS detoxification, and most, including a glutathione reductase, peroxidase, thaumatin, germin, and peroxiredoxin-5-like protein, were up-regulated (Table S5) further highlighting the importance of ROS scavenging systems inside and outside the cell.^69,70,72^ Our results collectively support the role of ABA in enhancing plant signaling, defense and/or ROS scavenging capacities during stress response.

We also identified nine glycoside hydrolase family 18 and 19 proteins in the secretome, and all were up-regulated, with fold-changes as high as 4.8 (Table S6). Plant glucoside hydrolases metabolize various carbohydrates involved in plant cell growth, signaling and response to biotic and abiotic stresses.^77,78^ Members of glycoside hydrolase family 18 and 19 possess chitinase activity, and several from Arabidopsis and rice are secreted proteins.^78^ Chitinases degrade chitin and are produced in response to pathogen attack, drought, salt, cold, wounding, heavy metal toxicity, and phytohormones such as ethylene, jasmonic acid and salicylic acid.^78–80^ For example, extracellular chitinases hydrolyze chitin of fungal origin, thus retarding hyphal growth and progression of fungus colonization in plants.^78,79^ These apoplastic chitinases may also participate in cell signaling as their chitin degradation products signal plant cells to mount further defense responses against the invading pathogen.^79^ Due to their induction following pathogen attack, chitinases are classified as pathogenesis-related proteins.^71^ Other plant secretome studies have identified increased accumulation of chitinases in response to heat,^48,81^ osmotic,^51^ salt^76^ and dehydration^82^ stresses. Our results also add to the growing literature on the role of ABA in inducing plant chitinases, possibly as a defense mechanism against environmental stresses.

#### Cell wall modifying enzymes

Plant cell walls are dynamic structures that dominate the ECM and are composed of cellulose microfibrils, hemicellulose, pectin, and proteins.^58,83,84^ Functionally, cell walls provide structural support to plant organs, maintain cell shape, offer a protective barrier against pathogen invasion and other external molecules, and are sites for cell-cell communication and signaling.^85–88^ As such, cell walls may undergo various modifications in structure and function in response to biotic and abiotic stresses.^88–90^ For example, cell wall strengthening or loosening occurs in response to various stresses, including pathogen attack, water deficits and salinity, and these processes are regulated by peroxidases, ROS, expansins and xyloglucan endotransglycosylases (XETs).^83,87–89^

The results of this study revealed that cell wall modifying enzymes (27%) were the second largest ABA-responsive functional group in the ECM as opposed to 15% in the TSP (Figure 3; Tables S5 and S6). Examples of the identified cell wall modifying enzymes in the ECM include expansins, pectinesterases, fasciclin-like arabinogalactan proteins, xyloglucan endotransglucosylase/hydrolase, and several members of glycoside hydrolases (Tables S5 and S6). About two-thirds of these cell wall modification-related secreted proteins, including expansins, glycoside hydrolases, and XETs, were up-regulated (Table S6). Expansins are cell wall-loosening proteins without lytic activity and have cell growth functions during plant development and in response to low phosphorus, drought, heat, and oxidative stresses.^91–93^ Likewise, xyloglucan modification by XETs enhances cell extensibility,^87^ and ABA is believed to regulate XET activity.^94^ Under water-limiting conditions, expansin and XET-mediated cell wall loosening enhance root growth for increased water-foraging capacity.^83,87^ Other secreted proteins identified include glycoside hydrolases that metabolize diverse polysaccharide components of the cell walls, such as pectin, xylan, arabinan, galactomannan, cellulose, glucan, and glycosaminoglycan (Table S6), further implicating ABA in plant cell wall biology. Numerous cell wall modifying enzymes have also been identified in other secretome studies in response to osmotic,^51^ and heat,^48,81^ dehydration^75,82^ stresses, thus emphasizing the pivotal roles of cell walls and their remodeling during stress response. A review by Albene et al.^90^ summarizes Arabidopsis cell wall proteomics studies, the major classes of cell wall protein families, and their interacting partners in cell walls.

#### Proteolysis

Proteolysis-related proteins were highly represented in the ABA-responsive secretome with 12 proteins (15%) but only two in the TSP (4%) (Figure 3; Tables S5 and S6). Both TSP proteins were proteasome subunits of the ubiquitin-proteasome system that degrades proteins in the cytosol.^95^ In contrast, the secretome exhibited greater diversity of aspartic, cysteine and serine-type endopeptidases and their inhibitors, such as cystatins, a Bowman-Birk type wound-induced proteinase inhibitor WIP1, and a proteinase inhibitor II3, potato inhibitor I (Table S6). All except the proteinase inhibitor II3, potato inhibitor I possessed signal peptides, indicating that secretory proteases and protease inhibitors respond to ABA. In addition, nine of these 12 proteins were up-regulated (Table S6). Our recent review^96^ and references therein project proteases and their inhibitors as critical role players in numerous physiological processes under normal growth and during stress response. Furthermore, transgenic studies using gain or loss-of-function mutants implicate proteases and/or protease inhibitors in ABA signaling processes.^96^ Apoplastic proteases are also regarded as defense systems against plant pathogen infection.^97,98^

#### Cellular transport

Cellular transport is essential for the trafficking of proteins, lipids, and other molecules within and between cells for various functions. Only one protein transporting GTPase was identified in the intracellular fraction and down-regulated (Table S5). In contrast, three lipid-transporting plant nonspecific lipid-transfer protein/Par allergens were identified in the secretome, and two were up-regulated, with fold-changes of 2.41 and 4.83 (Table S6). Plant nonspecific lipid-transfer protein/Par allergens are small, basic proteins with a wide range of functions, including lipid binding and transport.^99^ Their physiological roles are also diverse during plant growth and development and in response to biotic and abiotic stresses, including the thickening of the cuticle.^99^ Dani et al.^76^ also identified two salt-inducible lipid transfer proteins in the tobacco leaf apoplastic proteome following salt stress, suggesting their role is salt-response. Our results also suggest the implication of ABA in lipid transport processes in the sorghum ECM.

### Exogenous ABA regulates a broad spectrum of intracellular proteins involved in metabolism

The ABA-responsive TSP consisted of a broader selection of proteins involved in the metabolism of carbohydrates, amino acids, lipids and fatty acids, nitrogenous compounds, pigments, and lignin (Table S5). In addition, the metabolism functional group dominated the ABA-responsive TSP (43%) (Figure 3). Enzymes involved in the carbon fixation (phosphoenolpyruvate carboxylase) and glycolysis (pyruvate kinase, fructose-bisphosphate aldolase) were down-regulated (Table S5), possibly to save energy for other crucial stress-adaptive processes. Conversely, some enzymes involved in the metabolism of lipids and fatty acids (patatin, thiolase, and beta-hydroxyacyl-(acyl-carrier-protein) dehydratase FabZ), and lignin (cinnamyl alcohol dehydrogenase-like) were up-regulated (Table S5). In contrast, the metabolism-related functional group constituted 11% of the ABA-responsive secretome, and most were down-regulated (Figure 3). However, no theme could be drawn from the affected secreted proteins. Nevertheless, our results support the pivotal role of ABA in modulating stress metabolism of primary and secondary metabolites within and outside the cell.

### Signal transduction, protein synthesis, and DNA replication are unique to the intracellular proteome

The signal transduction, protein synthesis, and DNA replication categories were exclusively present in the ABA-responsive TSP (Figure 3; Tables S5 and S6). Both Bet v I type allergens involved in the ABA-activated signaling pathway were up-regulated (Table S5). Stress sensing and signaling are critical processes that precede other plant responses to environmental changes. The Bet v I protein is a birch pollen allergen,^100^ encoded by a gene family like the PYR/PRL-1/RCARs.^3,101,102^ As discussed earlier, PYR/PRL-1/RCARs are ABA receptors which bind ABA, freeing the SnRK2s from the inhibitory effects of PP2Cs. Subsequently, ABA-regulated gene expression changes are deployed, resulting in changes in cellular metabolism. Furthermore, GO annotations on the UniProt database suggest that both Bet v I type allergen proteins (accessions C5WMM0 and Q4VQB4) have molecular functions related to signal receptor activity, ABA binding, and protein phosphatase inhibitor activity (Table S5), which are similar to the functions of PYR/PRL-1/RCARs.^3^ The up-regulation of both Bet v I type allergen proteins in the current study (Table S5) further reinforces the positive interaction between ABA and its receptors in inactivating PP2Cs during ABA-dependent stress response.^101^

The up-regulation of the proliferating cell nuclear antigen (PCNA) in the intracellular fraction (Table S5) also points to the role of ABA in DNA replication. A review by Strzalka and Ziemienowicz^103^ extensively details various functions of PCNA in DNA replication, DNA repair and cell cycle control, which are vital for proper cell functioning. While molecular responses to ABA and various stresses involve global changes in protein synthesis, we identified threonine-tRNA ligase class IIa as the only protein synthesis-related protein, and it was down-regulated (Table S5). It is plausible that the duration of ABA treatment may influence the types of proteins identified and their expression levels. However, time-course proteomics experiments are required to investigate the changes in the types of proteins and their accumulation patterns as a function of ABA treatment duration.

Finally, the functions of 13 ABA-responsive proteins were unclear and thus grouped as unclassified proteins (Tables S5 and S6; Figure 3). Of these proteins, 10 were identified in the secretome fraction (Table S6), possibly indicating the pool of unknown sorghum ECM proteins that may have essential roles in stress adaptation. For example, leucine-rich repeat-containing N-terminal plant-type domain-containing proteins have been repeatedly identified as up-regulated proteins in response to drought in the intracellular proteome of sorghum roots,^52^ and osmotic^51^ and heat^48^ stresses in the sorghum secretome. Therefore, such unclassified proteins may represent gene products with universal roles in abiotic stress responses that await functional validation.

### The complex nature of ABA responses in the ECM versus the intracellular space

We also identified eight ABA-responsive proteins common in intracellular and secreted protein fractions (Table S7). Since all plant proteins are synthesized intracellularly before translocation to various destinations within or external to the cell, the common proteins could have been extracted before their total secretion. Alternatively, the proteins may have dual subcellular locations and/or multiple functions in plants. Furthermore, the expression levels of four of these proteins, two glycoside hydrolases (C5XKE9 and C5WXC7), a phytocyanin-like protein (C5YK12), and thaumatin (C5XN52) were statistically different between the two fractions (Table S7). As such, cellular localization and functional studies would possibly validate their subcellular location(s) and function(s) in each cell compartment and thus unravel the significance of their differential expression levels between the intracellular and extracellular fractions.

### Exogenous ABA down-regulates a myriad of ECM and intracellular proteins

When faced with stress, plants tend to slowdown or stop growth processes in order to redirect energy toward stress-adaptive processes and/or ration available nutrient resources for survival.^58,104,105^ The visible cessation of growth, as seen under drought and other abiotic stresses^105^ is underpinned by a reprogramming of metabolite flux across the entire network of metabolic circuitry.^104^ While post-translational modifications are key regulatory switches controlling enzyme activity, the downregulation of protein expression serves as a powerful strategy to tone-down or stop flux through selected metabolic pathways. We observed that 43% of the ABA-responsive ECM proteins was down-regulated and the majority were associated with metabolism, cell wall modification and defence/detoxification processes (Table S6). In the TSP, down-regulated proteins constituted 35% of the ABA-responsive proteins and were mainly associated with metabolism and defence/detoxification (Table S5). However, we could not discern any precise theme from the downregulated proteins (Tables S5 and S6). Nevertheless, it is possible the downregulation of protein expression observed in this study might represent aspects of constricting or shutting down certain metabolic pathways. In a way, this may contribute to induction of dormancy, which is necessary until the stress in relieved.^58^ We thus propose a global systems biology analysis to link these decreases in protein abundance with downstream changes in metabolite content and morpho-physiological properties of sorghum plants under ABA treatment with and without water deficit stress.

### Genes encoding proteins responsive to ABA *in vitro* also respond to drought *in planta*

**Figure 4.**
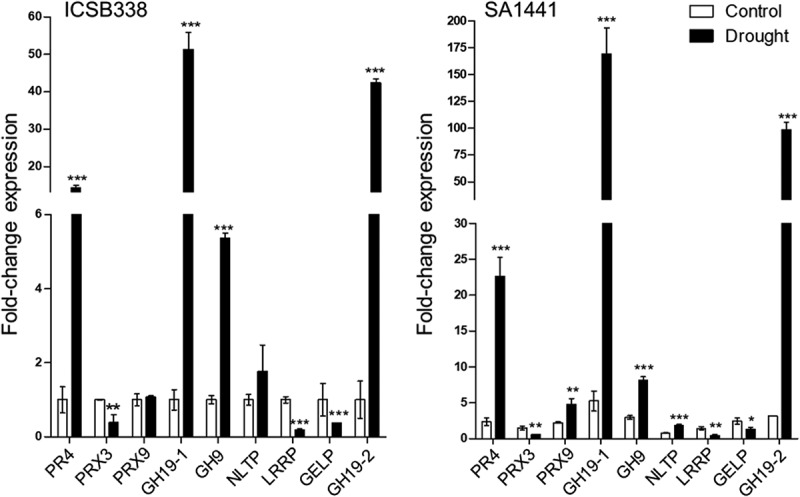
Gene expression analysis of sorghum root tissue following drought stress. Drought-susceptible ICSB338 and drought-tolerant SA1441 sorghum plants were exposed to drought stress by withholding water for 12 days. Root tissue samples were harvested for gene expression analysis using quantitative reverse transcription – polymerase chain reaction. Bars represent mean ± SD (*n* = 3). *, ** and *** represent statistical significance at *p* ≤.05, 0.01 and 0,001, respectively using a Student’s *t*-test.

The above results show the proteomic response of an *in vitro* sorghum cell culture system to the stress hormone ABA. Our hypothesis is that this provides new insights into what happens in plants responding to drought stress. To test this hypothesis, we used two previously characterized sorghum varieties^52^ with distinct drought response phenotypes – SA1441 (drought-tolerant) and ICSB338 (drought-susceptible) and investigated gene expression in drought-stressed root tissue. We selected nine genes encoding ABA-responsive proteins we identified in this study (Tables S5 and S6). This list consisted of up-regulated and down-regulated proteins: PR4 (SORBI_3005G169300), PRX3 (SORBI_3002G391300), PRX9 (SORBI_3003G152100), GH19–1 (SORBI_3006G132300), GH19–2 (SORBI_3006G132400), GH9 (SORBI_3003G015700), NLTP (SORBI_3008G030900), LRRP (SORBI_3005G126200) and GELP (SORBI_3010G044500). We found that drought activated the root tissue expression of all genes coding for proteins that were up-regulated by ABA in the cell culture system, except for SORBI_3010G044500, while genes encoding down-regulated proteins were also down-regulated (Figure 4). The magnitude of response for genes encoding up-regulated proteins was significantly higher in the drought-tolerant SA1441 sorghum variety than the susceptible ICSB338 variety. In fact, PRX9 and NLTP were not significantly up-regulated in ICSB338 variety, while in SA1441 the up-regulation was statistically significant. Thus, in addition to validating the proteome data, these results also show that the response is at the transcriptional level. More importantly, the results validate the *in vitro* cell culture system and confirms that ABA-induced responses are recapitulated in plants responding to drought.

## Conclusion

The phytohormone ABA^1,3,4^ and the plant ECM^40–43^ play key roles during stress-adaptive responses, yet our knowledge of the impact of ABA on secreted proteins is minimal. We observed a greater proportion of sorghum-secreted proteins (~32%) that were responsive to ABA than intracellular proteins (~7%), suggesting that the ECM could be an important target of ABA during stress-adaptive responses. The three most dominant groups in the ECM proteome were defense/detoxification (32%), cell wall modification (27%), and proteolysis (15%), suggesting that ABA drives plant defense and redox homeostasis, cell wall remodeling and protein degradation in the ECM. Our results suggest that depending on the prevailing stress condition promoting ABA accumulation, ABA-dependent responses could trigger diverse changes in cell metabolism to protect the cells from further stress damage. For example, the plant may stiffen its cell walls to impede total colonization by the invading pathogens or utilize various pathogenesis-related proteins (e.g. peroxidases, chitinases, RNA nucleases, thaumatins, and proteases) for diverse defense activities against the pathogen. Likewise, ABA-regulated cell wall loosening under drought or salinity stress may promote increased cell growth and root elongation to avoid stressful conditions. With such diverse effects of ABA on the secretome, we propose more transgenic studies to validate the roles of these ABA-responsive secreted proteins in susceptible plant species exposed to individual biotic and abiotic stresses and their combinations.

## Supplementary Material

Supplemental Material
