# Supplementary material for: Exogenous abscisic acid treatment regulates protein secretion in sorghum cell suspension cultures
Source: Plant Signal Behav. 2023 Dec 15;18(1):2291618. doi: 10.1080/15592324.2023.2291618 (PMC10730228; doi:10.1080/15592324.2023.2291618)
Supplement: Supplemental Material [file KPSB_A_2291618_SM6643.zip › Table S5.docx]

**Table S5.** List of ABA-responsive total soluble proteins of white sorghum cell suspension cultures at 5% significance level.

| **N^a^** | **Accession^b^** | **Protein name** | **Ratio^c^** | **SD^d^** | **p-value^e^** | **MW (kDa)^f^** | **SP^g^** | **Cellular component^h^** | **Biological process^i^** | **Molecular function^j^** | **Protein family^k^** |
| --- | --- | --- | --- | --- | --- | --- | --- | --- | --- | --- | --- |
| **Metabolism** | | | | | | | | | | | |
| 31 | C5XFH6 | Fructose-bisphosphate aldolase OS=*Sorghum bicolor* GN=SORBI_3003G393900 | -1.26 | 0.13 | 2.83E-02 | 38.56 | - | Cytosol | Glycolytic process | Fructose-bisphosphate aldolase activity | Fructose-bisphosphate aldolase, Class-I |
| 36 | A0A194YGV9 | Uncharacterized protein OS=*Sorghum bicolor* GN=SORBI_3010G023700 | 1.23 | 0.18 | 4.74E-02 | 79.57 | - | Cytosol | Pentose-phosphate shunt | Transketolase activity | Transketolase family |
| 41 | C5XW45 | Glyceraldehyde-3-phosphate dehydrogenase OS=*Sorghum bicolor* GN=SORBI_3004G056400 | 1.28 | 0.18 | 2.12E-02 | 43.45 | - | None | Glucose metabolic process | Oxidoreductase activity | Glyceraldehyde-3-phosphate dehydrogenase, type 1 |
| 44 | A0A1B6PEZ5 | Uncharacterized protein OS=*Sorghum bicolor* GN=SORBI_3007G014700 | 1.15 | 0.11 | 4.45E-02 | 35.51 | - | Cytosol | Fructose metabolic process | Fructokinase activity | Carbohydrate kinase PfkB domain-containing protein |
| 51 | C5YU02 | Uncharacterized protein OS=*Sorghum bicolor* GN=SORBI_3008G083400 | 1.15 | 0.04 | 2.00E-02 | 52.73 | - | Cytoplasm | Amino acid biosynthetic process | Argininosuccinate synthase activity | Argininosuccinate synthase, type 1 subfamily |
| 81 | A0A194YT53 | Uncharacterized protein OS=*Sorghum bicolor* GN=SORBI_3004G345800 | 1.41 | 0.15 | 2.70E-03 | 48.17 | - | Peroxisome | Fatty acid beta-oxidation | Acyltransferase activity | Thiolase |
| 205 | C5YAI8 | Pyruvate kinase OS=*Sorghum bicolor* GN=SORBI_3006G267200 | -1.35 | 0.09 | 2.36E-02 | 55.36 | - | Cytoplasm | Glycolytic process | Pyruvate kinase activity | Pyruvate kinase |
| 323 | A0A194YRE9 | Glutamine synthetase OS=*Sorghum bicolor* GN=SORBI_3004G247000 | 1.33 | 0.23 | 4.76E-02 | 39.18 | - | Cytoplasm | Nitrogen compound metabolic process | Glutamate-ammonia ligase activity | Glutamine synthetase |
| 333 | A0A1Z5RB28 | Patatin OS=*Sorghum bicolor* GN=SORBI_3007G158800 | 1.35 | 0.21 | 1.67E-02 | 44.02 | - | None | Lipid metabolic process | Lipase activity | Patatin |
| 340 | C5XI18 | S-adenosylmethionine synthase OS=*Sorghum bicolor* GN=SORBI_3003G140000 | -1.37 | 0.05 | 1.07E-03 | 43.26 | - | Cytoplasm | S-adenosylmethionine biosynthetic process | Methionine adenosylmethionine activity | S-adenosylmethionine synthetase |
| 424 | C5YE18 | Uncharacterized protein OS=*Sorghum bicolor* GN=SORBI_3006G181300 | 1.82 | 0.28 | 4.99E-02 | 62.03 | - | None | Pigment biosynthetic process | Catechol oxidase activity | Polyphenol oxidase |
| 456 | C5Z513 | Uncharacterized protein OS=*Sorghum bicolor* GN=SORBI_3010G171800 | -1.31 | 0.09 | 6.98E-03 | 63.13 | + | Extracellular region | None | L-ascorbate oxidase activity | L-ascorbate oxidase, plants |
| 496 | C5X951 | Uncharacterized protein OS=*Sorghum bicolor* GN=SORBI_3002G167000 | -1.30 | 0.05 | 3.61E-02 | 110.18 | - | Cytoplasm | Carbon fixation | Phosphoenolpyruvate carboxylase activity | Phosphoenolpyruvate carboxylase |
| 525 | C5Z6F3 | Uncharacterized protein OS=*Sorghum bicolor* GN=SORBI_3010G080300 | -1.53 | 0.10 | 4.28E-02 | 48.32 | + | Cytoplasm | Amino acid metabolic process | Aminoacylase activity | N-acyl-L-amino-acid amidohydrolase |
| 552 | C5YGY0 | Uncharacterized protein OS=*Sorghum bicolor* GN=SORBI_3007G043400 | 1.65 | 0.29 | 3.99E-02 | 42.55 | - | Cytoplasm | Pentose-phosphate shunt | Transaldolase activity | Transaldolase type 1 |
| 573 | C5YK12 | Uncharacterized protein OS=*Sorghum bicolor* GN=SORBI_3007G100600 | 1.65 | 0.38 | 4.08E-02 | 19.92 | + | Membrane | Electron transport chain | Electron transfer activity | Phytocyanin-like |
| 577 | C5YIY2 | Uncharacterized protein OS=*Sorghum bicolor* GN=SORBI_3007G079600 | 1.27 | 0.16 | 3.35E-02 | 24.32 | - | Cytoplasm | Lipid metabolic process | Hydro-lyase activity | Beta-hydroxyacyl-(acyl-carrier-protein) dehydratase FabZ |
| 594 | Q94IP1 | Cinnamic acid 4-hydroxylase OS=*Sorghum bicolor* GN=C4H | -1.20 | 0.06 | 3.79E-02 | 57.12 | - | Membrane | Lignin metabolic process | Trans-cinnamate 4-monooxygenase activity | Cytochrome P450, E-class, group 1 |
| 596 | C5XRZ8 | Uncharacterized protein OS=*Sorghum bicolor* GN=SORBI_3004G296800 | 1.28 | 0.13 | 8.89E-03 | 25.76 | - | None | Nitrogen compound metabolic process | Hydro-lyase activity | Aconitase A/isopropylmalate dehydratase small subunit swivel domain-containing |
| 855 | A0A1W0W560 | Uncharacterized protein OS=*Sorghum bicolor* GN=SORBI_3002G195700 | 1.31 | 0.18 | 4.55E-02 | 43.77 | - | None | Lignin biosynthetic process | Oxidoreductase activity | Cinnamyl alcohol dehydrogenase-like |
| **Defence/Detoxification** | | | | | | | | | | | |
| 35 | A0A194YU12 | Uncharacterized protein OS=*Sorghum bicolor* GN=SORBI_3004G341200 | 1.41 | 0.26 | 2.76E-02 | 53.04 | - | Cytoplasm | Cell redox homeostasis | Glutathione-disulfide reductase (NADPH) activity | Glutathione reductase |
| 63 | A0A1B6PFE9 | Uncharacterized protein OS=*Sorghum bicolor* GN=SORBI_3007G038600 | 1.15 | 0.06 | 4.73E-02 | 53.89 | - | Cytoplasm | Response to cold | Oxidoreductase activity | FAD/NAD(P)-binding domain-containing protein |
| 70 | C5XIY1 | Peroxidase OS=*Sorghum bicolor* GN=SORBI_3003G152100 | 2.98 | 1.22 | 1.74E-02 | 37.71 | + | Extracellular region | Response to oxidative stress | Peroxidase activity | Plant peroxidase |
| 132 | C5WWQ2 | Uncharacterized protein OS=*Sorghum bicolor* GN=SORBI_3001G342600 | -1.58 | 0.07 | 9.51E-03 | 64.78 | - | None | Cellular oxidant detoxification | Thioredoxin-disulfide reductase activity | Thioredoxin domain-containing protein |
| 235 | C5XN52 | Uncharacterized protein OS=*Sorghum bicolor* GN=SORBI_3003G331700 | 1.39 | 0.21 | 2.17E-02 | 23.88 | + | None | Defense response | None | Thaumatin family |
| 243 | C5WNY4 | Uncharacterized protein OS=*Sorghum bicolor* GN=SORBI_3001G129700 | 1.33 | 0.20 | 2.54E-02 | 23.75 | + | Apoplast | None | Metal ion binding | Germin |
| 346 | C5XG44 | Uncharacterized protein OS=*Sorghum bicolor* GN=SORBI_3003G254300 | 1.31 | 0.21 | 4.88E-02 | 17.38 | - | Cytoplasm | Cell redox homeostasis | Peroxidase activity | Peroxiredoxin-5-like |
| 401 | C5Z4V3 | Uncharacterized protein OS=*Sorghum bicolor* GN=SORBI_3010G051100 | -1.17 | 0.11 | 4.16E-02 | 60.20 | + | Endoplasmic reticulum | Response to endoplasmic reticulum stress | Protein disulfide isomerase activity | Thioredoxin domain-containing protein |
| 600 | A0A1B6QFT1 | Peroxidase OS=*Sorghum bicolor* GN=SORBI_3002G392000 | -1.40 | 0.15 | 2.01E-02 | 40.53 | + | Extracellular region | Response to oxidative stress | Peroxidase activity | Plant peroxidase |
| **Signal transduction** | | | | | | | | | | | |
| 47 | C5WMM0 | Uncharacterized protein OS=*Sorghum bicolor* GN=SORBI_3001G400900 | 1.47 | 0.23 | 8.53E-03 | 17.06 | - | Nucleus | Abscisic acid-activated signalling pathway | Abscisic acid binding | Bet v I type allergen |
| 167 | Q4VQB4 | Pathogenesis-related protein 10c OS=*Sorghum bicolor* GN=PR10 | 1.55 | 0.34 | 2.21E-02 | 16.78 | - | Nucleus | Abscisic acid-activated signalling pathway | Abscisic acid binding | Bet v I type allergen |
| **Proteolysis** | | | | | | | | | | | |
| 378 | C5WNX2 | Proteasome subunit beta type OS=*Sorghum bicolor* GN=SORBI_3001G128400 | -1.26 | 0.07 | 4.58E-02 | 23.18 | - | Proteasome complex | Proteolysis involved in protein catabolic process | None | Proteasome subunit beta 2 |
| 561 | C5Z3R9 | Proteasome subunit beta type OS=*Sorghum bicolor* GN=SORBI_3010G029400 | 1.30 | 0.04 | 6.25E-04 | 26.28 | - | Proteasome complex | Proteolysis involved in protein catabolic process | Threonine-type endopeptidase activity | Peptidase T1A, Proteasome beta-subunit |
| **Cell wall modification** | | | | | | | | | | | |
| 76 | C5XKE9 | Endoglucanase OS=*Sorghum bicolor* GN=SORBI_3003G015700 | 1.55 | 0.14 | 8.60E-04 | 69.78 | + | Extracellular region | Cellulose catabolic process | Hydrolase activity, hydrolyzing O-glycosyl compounds | Glycoside hydrolase family 9 |
| 113 | A0A1W0VUE2 | Uncharacterized protein OS=*Sorghum bicolor* GN=SORBI_3010G227400 | 1.26 | 0.12 | 1.74E-02 | 92.59 | - | None | Carbohydrate metabolic process | Hydrolase activity, hydrolyzing O-glycosyl compounds | Glycoside hydrolase family 31 |
| 181 | C5WXC7 | Alpha-galactosidase OS=*Sorghum bicolor* GN=SORBI_3001G208100 | -1.36 | 0.09 | 2.10E-02 | 46.96 | + | Plant-type cell wall | Galactomannan catabolic process | Hydrolase activity, hydrolyzing O-glycosyl compounds | Glycoside hydrolase, family 27 |
| 268 | C5WV02 | Uncharacterized protein OS=*Sorghum bicolor* GN=SORBI_3001G033300 | 1.32 | 0.16 | 4.58E-02 | 28.16 | + | Extracellular region | Cell wall organization | None | Expansin |
| 308 | C5WY32 | Uncharacterized protein OS=*Sorghum bicolor* GN=SORBI_3001G061900 | 1.32 | 0.16 | 3.05E-02 | 53.62 | + | Membrane | Carbohydrate metabolic process | Hydrolase activity, hydrolyzing O-glycosyl compounds | Glycoside hydrolase family 17 |
| 423 | C5WSF9 | Uncharacterized protein OS=*Sorghum bicolor* GN=SORBI_3001G301500 | 1.32 | 0.17 | 1.70E-02 | 30.89 | + | Extracellular region | Cell wall organization | None | Expansin |
| 571 | C5XVQ6 | Glycosyltransferase OS=*Sorghum bicolor* GN=SORBI_3004G191000 | 1.33 | 0.12 | 4.93E-03 | 56.01 | - | Cytoplasm | None | UDP-glycosyltransferase activity | UDP-glucuronosyl/UDP-glycosyltransferase |
| **Protein synthesis** | | | | | | | | | | | |
| 288 | C5WQD6 | Uncharacterized protein OS=*Sorghum bicolor* GN=SORBI_3001G145400 | -1.39 | 0.12 | 3.46E-02 | 84.47 | - | Mitochondrion | Translation | Nucleotide binding | Threonine-tRNA ligase, class IIa |
| **DNA replication** | | | | | | | | | | | |
| 160 | C5XV51 | Proliferating cell nuclear antigen OS=*Sorghum bicolor* GN=SORBI_3004G336600 | 1.27 | 0.12 | 2.61E-02 | 29.30 | - | Nucleus | Regulation of DNA replication | DNA polymerase processivity factor activity | Proliferating cell nuclear antigen, PCNA |
| **Cellular transport** | | | | | | | | | | | |
| 168 | C5XNL6 | Uncharacterized protein OS=*Sorghum bicolor* GN=SORBI_3003G189000 | -1.21 | 0.08 | 1.79E-02 | 22.52 | - | None | Vesicle-mediated transport | GTPase activity | Small GTPase |
| **Unclassified** | | | | | | | | | | | |
| 117 | C5XBP7 | Uncharacterized protein OS=*Sorghum bicolor* GN=SORBI_3002G343600 | 2.10 | 0.14 | 5.53E-05 | 35.66 | - | None | Specification of floral organ number | Enzyme inhibitor activity | Leucine-rich repeat-containing N-terminal plant-type domain-containing protein |
| 342 | C5X487 | Uncharacterized protein OS=*Sorghum bicolor* GN=SORBI_3002G111200 | -1.32 | 0.06 | 4.47E-02 | 26.72 | - | None | None | Oxidoreductase activity | Short-chain dehydrogenase/reductase SDR |
| 510 | C5X4M5 | Uncharacterized protein OS=*Sorghum bicolor* GN=SORBI_3002G255000 | -1.46 | 0.11 | 3.61E-02 | 25.95 | + | Membrane | None | None | DOMON domain-containing protein |

^a^Protein number (N) assigned in ProteinPilot.

^b^Protein accession numbers obtained from the UniProt database searches against sequences of *Sorghum bicolor* only.

^b^Ratio represents the average fold-change (*n* = 4) in response to ABA relative to the control. A positive value indicates up-regulation, while a negative value indicates down-regulation.

^d^Standard deviation of the fold-changes (*n* = 4).

^e^Probability value obtained from a Student’s *t*-test comparing the fold changes between the ABA treatment and the control (*n* = 4).

^f^Theoretical molecular weight (MW) of each protein as predicted by the Expasy Compute pI/Mw tool on the UniProt database (<https://uniprot.org>).

^g^Signal peptide (SP) prediction results for each protein as determined by the SignalP 6.0 server (<https://services.healthtech.dtu.dk/services/SignalP-6.0/>). + indicated presence of a signal peptide, while – indicates absence of a signal peptide.

^h-j^Gene Ontology terms for each protein as collated from the UniProt database.

^k^Family name as predicted using the InterPro (<http://www.ebi.ac.uk/interpro/>). In cases where protein families are not predicted, functional domains are listed instead.
