# Supplementary material for: Exogenous abscisic acid treatment regulates protein secretion in sorghum cell suspension cultures
Source: Plant Signal Behav. 2023 Dec 15;18(1):2291618. doi: 10.1080/15592324.2023.2291618 (PMC10730228; doi:10.1080/15592324.2023.2291618)
Supplement: Supplemental Material [file KPSB_A_2291618_SM6643.zip › Table S6.docx]

**Table S6.** List of ABA-responsive secreted proteins of white sorghum cell suspension cultures at 5% significance level.

| **N^a^** | **Accession^b^** | **Protein name** | **Ratio^c^** | **SD^d^** | **p-value^e^** | **MW (kDa)^f^** | **SP^g^** | **Cellular component^h^** | **Biological process^i^** | **Molecular function^j^** | **Protein family^k^** |
| --- | --- | --- | --- | --- | --- | --- | --- | --- | --- | --- | --- |
| **Metabolism** | | | | | | | | | | | |
| 15 | C5Z240 | Uncharacterized protein OS=*Sorghum bicolor* GN=SORBI_3010G003100 | -1.20 | 0.07 | 1.94E-02 | 65.57 | + | None | None | Copper ion binding | Multicopper oxidase |
| 41 | C5YK12 | Uncharacterized protein OS=*Sorghum bicolor* GN=SORBI_3007G100600 | -1.31 | 0.10 | 3.91E-02 | 19.92 | + | Membrane | Electron transport chain | Electron transfer activity | Phytocyanin-like |
| 43 | C5XWE5 | Uncharacterized protein OS=*Sorghum bicolor* GN=SORBI_3004G197600 | -1.29 | 0.04 | 1.62E-02 | 81.75 | + | None | Lipid metabolic process | Glycerophosphodiester phosphodiesterase activity | Glycerophosphodiester phosphodiesterase |
| 106 | C5Y9T3 | Aldose 1-epimerase OS=*Sorghum bicolor* GN=SORBI_3006G105200 | 1.42 | 0.25 | 2.07E-02 | 39.87 | + | None | Carbohydrate metabolic process | Aldose 1-epimerase activity | Aldose 1-epimerase |
| 107 | A0A1W0VY92 | Uncharacterized protein OS=*Sorghum bicolor* GN=SORBI_3003G205900 | -1.21 | 0.14 | 4.52E-02 | 39.38 | + | None | None | Hydrolase activity. acting on ester bonds | GDSL lipase/esterase-like. plant |
| 118 | A0A1B6Q4S6 | Uncharacterized protein OS=*Sorghum bicolor* GN=SORBI_3003G226300 | 1.30 | 0.06 | 1.71E-02 | 86.75 | + | Extracellular region | Lipid metabolic process | N-acylsphingosine amidohydrolase activity | Neutral/alkaline nonlysosomal ceramidase |
| 186 | C5Y1P6 | Uncharacterized protein OS=*Sorghum bicolor* GN=SORBI_3005G099500 | -1.61 | 0.16 | 1.04E-02 | 49.29 | + | Membrane | Nucleoside diphosphate catabolic process | Nucleoside diphosphate phosphatase activity | Nucleoside phosphatase GDA1/CD39 |
| 191 | C5Z4E5 | Uncharacterized protein OS=*Sorghum bicolor* GN=SORBI_3010G044900 | -1.68 | 0.09 | 6.64E-04 | 39.46 | + | None | None | Hydrolase activity. acting on ester bonds | GDSL lipase/esterase-like. plant |
| 208 | A0A194YIA9 | Uncharacterized protein OS=*Sorghum bicolor* GN=SORBI_3010G044500 | 2.92 | 0.44 | 2.22E-04 | 38.36 | + | None | None | Hydrolase activity. acting on ester bonds | GDSL lipase/esterase-like, plant |
| **Defence/Detoxification** | | | | | | | | | | | |
| 1 | C5Z475 | Peroxidase OS=*Sorghum bicolor* GN=SORBI_3010G162000 | 1.42 | 0.24 | 1.49E-02 | 34.43 | + | Extracellular region | Response to oxidative stress | Peroxidase activity | Plant Peroxidase |
| 5 | C5X5K6 | Peroxidase OS=*Sorghum bicolor* GN=SORBI_3002G416700 | -1.38 | 0.03 | 3.53E-03 | 32.41 | + | Extracellular region | Response to oxidative stress | Peroxidase activity | Plant Peroxidase |
| 6 | C5WYQ4 | Peroxidase OS=*Sorghum bicolor* GN=SORBI_3001G360400 | -1.65 | 0.09 | 3.82E-04 | 34.75 | + | Extracellular region | Response to oxidative stress | Peroxidase activity | Plant Peroxidase |
| 11 | C5Y1P4 | Uncharacterized protein OS=*Sorghum bicolor* GN=SORBI_3005G099000 | 1.60 | 0.32 | 1.07E-02 | 34.38 | + | Extracellular region | Carbohydrate metabolic process | Chitinase activity | GH18 domain-containing protein |
| 12 | C5Y360 | Peroxidase OS=*Sorghum bicolor* GN=SORBI_3005G011300 | -1.51 | 0.09 | 2.73E-02 | 34.43 | + | Extracellular region | Response to oxidative stress | Peroxidase activity | Plant Peroxidase |
| 19 | C5YBE9 | Uncharacterized protein OS=*Sorghum bicolor* GN=SORBI_3006G132400 | 3.93 | 0.31 | 1.73E-06 | 28.56 | + | None | Chitin catabolic process | Chitinase activity | Glycoside hydrolase family 19 |
| 25 | C5XB38 | Uncharacterized protein OS=*Sorghum bicolor* GN=SORBI_3002G055600 | 2.05 | 0.19 | 8.37E-05 | 33.65 | + | Extracellular region | Carbohydrate metabolic process | Chitinase activity | GH18 domain-containing protein |
| 26 | C5XCE2 | Uncharacterized protein OS=*Sorghum bicolor* GN=SORBI_3002G351400 | 1.63 | 0.27 | 6.06E-03 | 24.57 | + | None | Defense response | None | Thaumatin family |
| 27 | C5X3C1 | Peroxidase OS=*Sorghum bicolor* GN=SORBI_3002G391300 | -2.25 | 0.03 | 2.00E-02 | 31.10 | + | Extracellular region | Response to oxidative stress | Peroxidase activity | Plant peroxidase |
| 29 | C5XN52 | Uncharacterized protein OS=*Sorghum bicolor* GN=SORBI_3003G331700 | 2.21 | 0.22 | 4.79E-05 | 23.88 | + | None | Defense response | None | Thaumatin family |
| 33 | A0A1W0W7I8 | Peroxidase OS=*Sorghum bicolor* GN=SORBI_3002G391900 | -1.66 | 0.13 | 3.00E-02 | 57.58 | - | Plant-type cell wall | Response to oxidative stress | Peroxidase activity | Plant peroxidase |
| 34 | C5XB39 | Uncharacterized protein OS=*Sorghum bicolor* GN=SORBI_3002G055700 | 1.40 | 0.29 | 4.93E-02 | 33.60 | + | Extracellular region | Carbohydrate metabolic process | Chitinase activity | GH18 domain-containing protein |
| 35 | A0A1Z5RDM9 | Uncharacterized protein OS=*Sorghum bicolor* GN=SORBI_3006G132100 | 1.34 | 0.10 | 2.39E-02 | 24.56 | + | None | Chitin catabolic process | Chitinase activity | Glycoside hydrolase. family 19 |
| 51 | C5YBE8 | Uncharacterized protein OS=*Sorghum bicolor* GN=SORBI_3006G132300 | 4.81 | 0.89 | 1.62E-04 | 28.28 | + | None | Chitin catabolic process | Chitinase activity | Glycoside hydrolase. family 19 |
| 54 | C5YM54 | Uncharacterized protein OS=*Sorghum bicolor* GN=SORBI_3007G151300 | -1.93 | 0.02 | 7.23E-03 | 22.73 | + | Apoplast | None | Metal ion binding | Germin |
| 62 | A0A1W0VX32 | Peroxidase OS=*Sorghum bicolor* GN=SORBI_3003G127100 | -2.03 | 0.08 | 3.45E-02 | 35.93 | + | Extracellular region | Response to oxidative stress | Peroxidase activity | Plant Peroxidase |
| 74 | C5Y5V0 | Uncharacterized protein OS=*Sorghum bicolor* GN=SORBI_3005G177600 | 1.40 | 0.07 | 8.58E-03 | 32.94 | + | Extracellular region | Carbohydrate metabolic process | Chitinase activity | GH18 domain-containing protein |
| 84 | A0A109NDM1 | Uncharacterized protein (Fragment) OS=*Sorghum bicolor* GN=SORBI_3010G273600 | 1.76 | 0.55 | 3.50E-02 | 33.82 | + | None | Chitin catabolic process | Chitinase activity | Glycoside hydrolase. family 19 |
| 142 | C5YC92 | Uncharacterized protein OS=*Sorghum bicolor* GN=SORBI_3006G018100 | 1.43 | 0.08 | 5.84E-05 | 24.97 | + | Apoplast | None | Metal ion binding | Germin |
| 161 | C5YQ75 | Peroxidase OS=*Sorghum bicolor* GN=SORBI_3008G010500 | -1.85 | 0.11 | 2.75E-04 | 34.73 | + | Extracellular region | Response to oxidative stress | Peroxidase activity | Plant Peroxidase |
| 171 | C5Y5U9 | Uncharacterized protein OS=*Sorghum bicolor* GN=SORBI_3005G177500 | 1.24 | 0.07 | 6.06E-03 | 33.36 | + | Extracellular region | Carbohydrate metabolic process | Chitinase activity | GH18 domain-containing protein |
| 180 | C5Y5D5 | Uncharacterized protein OS=*Sorghum bicolor* GN=SORBI_3005G169300 | 2.68 | 0.58 | 1.40E-03 | 16.02 | + | None | Defense response | RNA nuclease activity | Pathogenesis-related protein-4 |
| 183 | A0A1B6QJR7 | Peroxidase OS=*Sorghum bicolor* GN=SORBI_3001G189000 | 1.80 | 0.35 | 3.98E-03 | 43.29 | - | Extracellular region | Response to oxidative stress | Peroxidase activity | Plant peroxidase |
| 216 | A0A1B6QN96 | Superoxide dismutase [Cu-Zn] OS=*Sorghum bicolor* GN=SORBI_3001G371900 | -1.80 | 0.16 | 2.39E-02 | 15.09 | - | None | Removal of superoxide radicals | Superoxide dismutase activity | Superoxide dismutase (Cu/Zn) / superoxide dismutase copper chaperone |
| 265 | C5XIY0 | Peroxidase OS=*Sorghum bicolor* GN=SORBI_3003G152000 | -1.49 | 0.05 | 7.51E-03 | 37.65 | + | Extracellular region | Response to oxidative stress | Peroxidase activity | Plant peroxidase |
| 269 | A0A1B6QG28 | Superoxide dismutase [Cu-Zn] OS=*Sorghum bicolor* GN=SORBI_3002G407900 | -1.59 | 0.26 | 3.49E-02 | 16.98 | - | None | Removal of superoxide radicals | Superoxide dismutase activity | Superoxide dismutase (Cu/Zn) / superoxide dismutase copper chaperone |
| **Proteolysis** | | | | | | | | | | | |
| 17 | C5XQ74 | Uncharacterized protein OS=*Sorghum bicolor* GN=SORBI_3003G208800 | 1.17 | 0.10 | 2.95E-02 | 53.53 | + | None | Proteolysis | Aspartic-type endopeptidase activity | Aspartic peptidase A1 family |
| 49 | C5Y675 | Uncharacterized protein OS=*Sorghum bicolor* GN=SORBI_3005G064200 | -1.30 | 0.09 | 8.37E-03 | 44.46 | + | None | Proteolysis | Aspartic-type endopeptidase activity | Aspartic peptidase A1 family |
| 82 | C5WVG9 | Cysteine proteinase inhibitor OS=*Sorghum bicolor* GN=SORBI_3001G324800 | -1.58 | 0.09 | 3.44E-03 | 14.38 | + | None | Negative regulation of peptidase activity | Cysteine-type endopeptidase inhibitor activity | Cystatin |
| 86 | C5XHP7 | Uncharacterized protein OS=*Sorghum bicolor* GN=SORBI_3003G419300 | 1.57 | 0.40 | 4.95E-02 | 44.95 | + | None | Proteolysis | Aspartic-type endopeptidase activity | Aspartic peptidase A1 family |
| 117 | A0A1B6Q6M7 | Cysteine proteinase inhibitor OS=*Sorghum bicolor* GN=SORBI_3003G327700 | 1.91 | 0.26 | 7.40E-04 | 14.89 | + | None | Negative regulation of peptidase activity | Cysteine-type endopeptidase inhibitor activity | Cystatin |
| 131 | C5XQP2 | Uncharacterized protein OS=*Sorghum bicolor* GN=SORBI_3003G078400 | 1.41 | 0.13 | 2.64E-02 | 52.20 | + | None | Proteolysis | Aspartic-type endopeptidase activity | Aspartic peptidase A1 family |
| 132 | A0A1B6P5R2 | Uncharacterized protein OS=*Sorghum bicolor* GN=SORBI_3009G009600 | 2.02 | 0.34 | 2.32E-03 | 16.56 | - | None | Negative regulation of peptidase activity | Serine-type endopeptidase inhibitor activity | Proteinase inhibitor I13. potato inhibitor I |
| 140 | C5YA35 | Uncharacterized protein OS=*Sorghum bicolor* GN=SORBI_3006G260300 | -1.28 | 0.06 | 1.53E-02 | 50.21 | + | Extracellular space | Proteolysis | Cysteine-type endopeptidase activity | Peptidase C1A |
| 164 | C5YPF6 | Uncharacterized protein OS=*Sorghum bicolor* GN=SORBI_3008G119900 | 1.71 | 0.45 | 2.53E-02 | 76.80 | + | None | Proteolysis | Serine-type endopeptidase activity | Subtilisin-like protease |
| 203 | A0A1B6Q242 | Uncharacterized protein OS=*Sorghum bicolor* GN=SORBI_3003G085300 | 1.64 | 0.48 | 4.85E-02 | 10.58 | + | Extracellular region | Negative regulation of peptidase activity | Serine-type endopeptidase inhibitor activity | Bowman-Birk type wound-induced proteinase inhibitor WIP1 |
| 226 | C5YNA1 | Uncharacterized protein OS=*Sorghum bicolor* GN=SORBI_3007G172100 | 1.47 | 0.10 | 1.33E-03 | 40.26 | + | Extracellular space | Proteolysis | Cysteine-type endopeptidase activity | Peptidase C1A |
| 235 | C5X0Y6 | Uncharacterized protein OS=*Sorghum bicolor* GN=SORBI_3001G529700 | 1.70 | 0.14 | 3.77E-04 | 79.49 | + | None | Proteolysis | Serine-type endopeptidase activity | Subtilisin-like protease |
| **Cell wall modification** | | | | | | | | | | | |
| 3 | C5XYP5 | Uncharacterized protein OS=*Sorghum bicolor* GN=SORBI_3004G233700 | -1.28 | 0.03 | 6.49E-04 | 84.26 | + | Extracellular region | Xylan catabolic process | Hydrolase activity. hydrolyzing O-glycosyl compounds | Beta-D-xylosidase |
| 9 | C5WXC7 | Alpha-galactosidase OS=*Sorghum bicolor* GN=SORBI_3001G208100 | -1.71 | 0.03 | 1.62E-02 | 46.96 | + | Plant-type cell wall | Galactomannan catabolic process | Hydrolase activity. hydrolyzing O-glycosyl compounds | Glycoside hydrolase. family 27 |
| 13 | C5Z8N0 | Uncharacterized protein OS=*Sorghum bicolor* GN=SORBI_3010G118900 | -1.56 | 0.06 | 2.95E-03 | 47.82 | + | Plasma membrane | None | None | Fasciclin-like arabinogalactan protein |
| 16 | C5XKE9 | Endoglucanase OS=*Sorghum bicolor* GN=SORBI_3003G015700 | 3.30 | 0.88 | 2.22E-03 | 69.78 | + | Extracellular region | Cellulose catabolic process | Hydrolase activity. hydrolyzing O-glycosyl compounds | Glycoside hydrolase family 9 |
| 22 | C5Z8T4 | Xyloglucan endotransglucosylase/hydrolase OS=*Sorghum bicolor* GN=SORBI_3010G246600 | -1.47 | 0.06 | 6.01E-05 | 31.49 | + | Extracellular region | Cell wall organization | Hydrolase activity. hydrolyzing O-glycosyl compounds | Xyloglucan endotransglucosylase/hydrolase |
| 44 | C5YVJ7 | Uncharacterized protein OS=*Sorghum bicolor* GN=SORBI_3009G232100 | -1.91 | 0.04 | 3.01E-02 | 24.90 | + | Plasma membrane | None | None | Fasciclin-like arabinogalactan protein. Group A |
| 50 | C5X022 | Uncharacterized protein OS=*Sorghum bicolor* GN=SORBI_3001G525000 | -1.58 | 0.01 | 3.90E-04 | 49.22 | + | None | Carbohydrate metabolic process | Hydrolase activity. hydrolyzing O-glycosyl compounds | Glycoside hydrolase. family 28 |
| 57 | C5XRX3 | Uncharacterized protein OS=*Sorghum bicolor* GN=SORBI_3004G294500 | 1.29 | 0.14 | 4.28E-02 | 27.41 | + | Extracellular region | Cell wall organization | None | Expansin |
| 64 | A0A1B6Q838 | Uncharacterized protein OS=*Sorghum bicolor* GN=SORBI_3003G422200 | 1.84 | 0.23 | 1.68E-03 | 34.95 | + | None | Carbohydrate metabolic process | Hydrolase activity. hydrolyzing O-glycosyl compounds | Glycoside hydrolase family 17 |
| 66 | A0A1B6QI05 | Uncharacterized protein OS=*Sorghum bicolor* GN=SORBI_3001G089100 | -1.26 | 0.12 | 2.45E-02 | 67.20 | + | None | Glucan catabolic process | Hydrolase activity. hydrolyzing O-glycosyl compounds | Beta-glucosidase |
| 71 | C5Z8T5 | Xyloglucan endotransglucosylase/hydrolase OS=*Sorghum bicolor* GN=SORBI_3010G246700 | 1.44 | 0.13 | 1.22E-02 | 33.42 | + | Extracellular region | Cell wall organization | Hydrolase activity. hydrolyzing O-glycosyl compounds | Xyloglucan endotransglucosylase/hydrolase |
| 85 | A0A1W0VUE2 | Uncharacterized protein OS=*Sorghum bicolor* GN=SORBI_3010G227400 | 1.28 | 0.17 | 4.70E-02 | 92.60 | - | None | Carbohydrate metabolic process | Hydrolase activity. hydrolyzing O-glycosyl compounds | Glycoside hydrolase. family 31 |
| 90 | C5WSE5 | Uncharacterized protein OS=*Sorghum bicolor* GN=SORBI_3001G300400 | 1.66 | 0.27 | 4.82E-03 | 31.85 | + | Extracellular region | Cell wall organization | None | Expansin |
| 127 | C5WSF9 | Uncharacterized protein OS=*Sorghum bicolor* GN=SORBI_3001G301500 | 1.62 | 0.41 | 4.54E-02 | 30.89 | + | Extracellular region | Cell wall organization | None | Expansin |
| 134 | A0A1B6QE21 | UTP--glucose-1-phosphate uridylyltransferase OS=*Sorghum bicolor* GN=SORBI_3002G291200 | 1.38 | 0.27 | 3.32E-02 | 52.25 | - | Cytoplasm | UDP-glucose metabolic process | UTP:glucose-1-phosphate uridylytransferase activity | UDP-glucuronosyl/UDP-glycosyltransferase |
| 155 | A0A1B6Q7A6 | Pectinesterase OS=*Sorghum bicolor* GN=SORBI_3003G376900 | 1.31 | 0.22 | 4.36E-02 | 57.42 | + | Extracellular region | Cell wall organization | Pectinesterase activity | Pectinesterase |
| 178 | C5WSY5 | Uncharacterized protein OS=*Sorghum bicolor* GN=SORBI_3001G014700 | -1.72 | 0.16 | 4.95E-02 | 51.21 | + | None | Carbohydrate metabolic process | Hydrolase activity. hydrolyzing O-glycosyl compounds | Glycoside hydrolase family 17. plant |
| 188 | C5XHS1 | Uncharacterized protein OS=*Sorghum bicolor* GN=SORBI_3003G422000 | 2.58 | 0.43 | 5.39E-03 | 35.69 | + | None | Carbohydrate metabolic process | Hydrolase activity. hydrolyzing O-glycosyl compounds | Glycoside hydrolase family 17. plant |
| 225 | C5XIT5 | Pectinesterase OS=*Sorghum bicolor* GN=SORBI_3003G148300 | -1.53 | 0.06 | 5.68E-03 | 59.38 | + | Extracellular region | Cell wall organization | Pectinesterase activity | Pectinesterase |
| 236 | C5WV02 | Uncharacterized protein OS=*Sorghum bicolor* GN=SORBI_3001G033300 | 1.52 | 0.16 | 3.64E-02 | 28.16 | + | Extracellular region | Cell wall organization | None | Expansin |
| 221 | C5XHR8 | Uncharacterized protein OS=*Sorghum bicolor* GN=SORBI_3003G421700 | 1.55 | 0.29 | 1.04E-02 | 34.56 | + | None | Carbohydrate metabolic process | Hydrolase activity. hydrolyzing O-glycosyl compounds | Glycoside hydrolase family 17. plant |
| 229 | C5XCD4 | Beta-hexosaminidase OS=*Sorghum bicolor* GN=SORBI_3002G350700 | 1.44 | 0.25 | 3.79E-02 | 63.80 | + | Membrane | Carbohydrate metabolic process | Hydrolase activity. hydrolyzing O-glycosyl compounds | Beta-hexosaminidase |
| **Cellular transport** | | | | | | | | | | | |
| 14 | A0A1Z5R5E6 | Non-specific lipid-transfer protein OS=*Sorghum bicolor* GN=SORBI_3008G030900 | 4.83 | 2.07 | 1.02E-02 | 11.54 | + | None | Lipid transport | Lipid binding | Plant non-specific lipid-transfer protein/Par allergen |
| 105 | C5YRL0 | Non-specific lipid-transfer protein OS=*Sorghum bicolor* GN=SORBI_3008G030700 | 2.41 | 0.54 | 2.19E-03 | 12.05 | + | None | Lipid transport | Lipid binding | Plant non-specific lipid-transfer protein/Par allergen |
| 213 | C5XAF8 | Uncharacterized protein OS=*Sorghum bicolor* GN=SORBI_3002G050400 | -1.43 | 0.19 | 2.85E-02 | 17.23 | + | None | Lipid transport | Lipid binding | Plant non-specific lipid-transfer protein/Par allergen |
| **Unclassified** | | | | | | | | | | | |
| 42 | C5XBP7 | Uncharacterized protein OS=*Sorghum bicolor* GN=SORBI_3002G343600 | 1.82 | 0.35 | 4.37E-03 | 35.66 | + | None | Specification of floral organ number | Protein binding | Leucine-rich repeat-containing N-terminal plant-type domain-containing protein |
| 55 | C5YBH7 | Uncharacterized protein OS=*Sorghum bicolor* GN=SORBI_3006G135500 | -1.43 | 0.05 | 1.14E-02 | 60.81 | + | None | None | None | Galactose oxidase-like Early set domain-containing protein |
| 61 | C5Z6D9 | Uncharacterized protein OS=*Sorghum bicolor* GN=SORBI_3010G079100 | -1.31 | 0.03 | 4.06E-02 | 40.67 | + | None | None | None | LysM domain-containing protein |
| 81 | C5XYB4 | Uncharacterized protein OS=*Sorghum bicolor* GN=SORBI_3004G229300 | 2.36 | 0.28 | 3.93E-03 | 34.29 | + | Extracellular space | None | None | Protein EXORDIUM-like |
| 103 | C5XL56 | Uncharacterized protein OS=*Sorghum bicolor* GN=SORBI_3003G023800 | 1.48 | 0.33 | 4.50E-02 | 61.96 | + | None | None | None | Peptide-N4-(N-acetyl-beta-glucosaminyl)asparagine amidase A |
| 116 | C5WPH7 | Uncharacterized protein OS=*Sorghum bicolor* GN=SORBI_3001G131100 | -1.27 | 0.05 | 1.84E-02 | 75.94 | + | None | None | None | Glucose/Sorbosone dehydrogenase domain-containing protein |
| 137 | C5Z6Y0 | Uncharacterized protein OS=*Sorghum bicolor* GN=SORBI_3010G088700 | 2.73 | 0.22 | 1.35E-05 | 34.48 | + | Extracellular space | None | None | Protein EXORDIUM-like |
| 150 | A0A1Z5RBA4 | Uncharacterized protein OS=*Sorghum bicolor* GN=SORBI_3007G225400 | 2.59 | 0.84 | 1.04E-02 | 32.57 | + | Extracellular space | None | None | Protein EXORDIUM-like |
| 173 | C5YI64 | Uncharacterized protein OS=*Sorghum bicolor* GN=SORBI_3007G198000 | -1.56 | 0.14 | 7.04E-03 | 25.17 | + | Membrane | None | None | DOMON domain-containing protein |
| 281 | C5Y2R8 | Uncharacterized protein OS=*Sorghum bicolor* GN=SORBI_3005G126200 | -3.34 | 0.10 | 9.82E-03 | 26.58 | + | None | None | Protein binding | Leucine-rich repeat-containing N-terminal plant-type domain-containing protein |

^a^Protein number (N) assigned in ProteinPilot.

^b^Protein accession numbers obtained from the UniProt database searches against sequences of *Sorghum bicolor* only.

^b^Ratio represents the average fold-change (*n* = 4) in response to ABA relative to the control. A positive value indicates up-regulation, while a negative value indicates down-regulation.

^d^Standard deviation of the fold-changes (*n* = 4).

^e^Probability value obtained from a Student’s *t*-test comparing the fold changes between the ABA treatment and the control (*n* = 4).

^f^Theoretical molecular weight (MW) of each protein as predicted by the Expasy Compute pI/Mw tool on the UniProt database (<https://uniprot.org>).

^g^Signal peptide (SP) prediction results for each protein as determined by the SignalP 6.0 server (<https://services.healthtech.dtu.dk/services/SignalP-6.0/>). + indicated presence of a signal peptide, while – indicates absence of a signal peptide.

^h-j^Gene Ontology terms for each protein as collated from the UniProt database.

^k^Family name as predicted using the InterPro (<http://www.ebi.ac.uk/interpro/>). In cases where protein families are not predicted, functional domains are listed instead.
