# Supplementary material for: Exogenous abscisic acid treatment regulates protein secretion in sorghum cell suspension cultures
Source: Plant Signal Behav. 2023 Dec 15;18(1):2291618. doi: 10.1080/15592324.2023.2291618 (PMC10730228; doi:10.1080/15592324.2023.2291618)
Supplement: Supplemental Material [file KPSB_A_2291618_SM6643.zip › Table S7.docx]

**Table S7.** List of ABA-responsive sorghum proteins that are common to both the total soluble proteome and the secretome.

| **Accession^a^** | **Protein name** | **Total soluble proteins** | | | **Secreted proteins** | | | **Total soluble vs. secreted proteins** | **Protein family^f^** |
| --- | --- | --- | --- | --- | --- | --- | --- | --- | --- |
|  |  | **Ratio^b^** | **SD^c^** | **p-value^d^** | **Ratio^b^** | **SD^c^** | **p-value^d^** | **Ratio p-value^e^** |  |
| **Metabolism** | | | | | | | | | |
| C5YK12 | Uncharacterized protein OS=*Sorghum bicolor* GN=SORBI_3007G100600 | 1.65 | 0.38 | 4.08E-02 | -1.31 | 0.10 | 3.91E-02 | 4.07E-03^*^ | Phytocyanin-like |
| **Defence/Detoxification** | | | | | | | | | |
| C5XN52 | Uncharacterized protein OS=*Sorghum bicolor* GN=SORBI_3003G331700 | 1.39 | 0.21 | 2.17E-02 | 2.21 | 0.22 | 4.79E-05 | 1.80E-03^*^ | Thaumatin family |
| **Cell wall modification** | | | | | | | | | |
| C5WV02 | Uncharacterized protein OS=*Sorghum bicolor* GN=SORBI_3001G033300 | 1.32 | 0.16 | 4.58E-02 | 1.52 | 0.16 | 3.64E-02 | 1.17E-01 | Expansin |
| C5WSF9 | Uncharacterized protein OS=*Sorghum bicolor* GN=SORBI_3001G301500 | 1.32 | 0.17 | 1.70E-02 | 1.62 | 0.41 | 4.54E-02 | 2.21E-01 | Expansin |
| C5XKE9 | Endoglucanase OS=*Sorghum bicolor* GN=SORBI_3003G015700 | 1.55 | 0.14 | 8.60E-04 | 3.30 | 0.88 | 2.22E-03 | 7.69E-03^*^ | Glycoside hydrolase family 9 |
| A0A1W0VUE2 | Uncharacterized protein OS=*Sorghum bicolor* GN=SORBI_3010G227400 | 1.26 | 0.12 | 1.74E-02 | 1.28 | 0.17 | 4.70E-02 | 8.69E-01 | Glycoside hydrolase family 31 |
| C5WXC7 | Alpha-galactosidase OS=*Sorghum bicolor* GN=SORBI_3001G208100 | -1.36 | 0.09 | 2.10E-02 | -1.71 | 0.03 | 1.62E-02 | 1.93E-02^*^ | Glycoside hydrolase. family 27 |
| **Unclassified** | | | | | | | | | |
| C5XBP7 | Uncharacterized protein OS=*Sorghum bicolor* GN=SORBI_3002G343600 | 2.10 | 0.14 | 5.53E-05 | 1.82 | 0.35 | 4.37E-03 | 1.99E-01 | Leucine-rich repeat-containing N-terminal plant-type domain-containing protein |

^a^Protein accession numbers obtained from the UniProt database searches against sequences of *Sorghum bicolor* only.

^b^Ratio represents the average fold-change (*n* = 4) in response to ABA relative to the control within each proteome. A positive value indicates up-regulation, while a negative value indicates down-regulation.

^c^Standard deviation of the fold-changes (*n* = 4) for each proteome.

^d^Probability value obtained from a Student’s *t*-test comparing the fold changes between the ABA treatment and the control (*n* = 4) within each proteome.

^e^Probability value obtained from a Student’s *t*-test comparing the fold changes between biological replicates of the total soluble proteome and the secreted proteome (*n* = 4). The proteins marked with an asterisk (*) had fold changes that were significantly different between the two sorghum proteomes.

^f^Family name as predicted using the InterPro (<http://www.ebi.ac.uk/interpro/>). In cases where protein families are not predicted, functional domains are listed instead.
